# Geospatial estimates of suicidal ideation and suicide attempt prevalence in the U.S. veteran population (2022)

**DOI:** 10.1186/s40621-025-00584-y

**Published:** 2025-06-10

**Authors:** Julie A. Kittel, Lindsey L. Monteith, Ryan Holliday, Theresa T. Morano, Alexandra L. Schneider, Lisa A. Brenner, Claire A. Hoffmire

**Affiliations:** 1 VA Rocky Mountain Mental Illness Research, Education and Clinical Center for Suicide Prevention, Aurora, CO USA; 2 Department of Physical Medicine and Rehabilitation, University of Colorado Anschutz Medical Campus, Aurora, CO USA; 3 Spark M. Matsunaga VA Medical Center, VA Pacific Islands Healthcare System, Honolulu, HI USA; 4 Department of Psychiatry, University of Colorado Anschutz Medical Campus, Aurora, CO USA

**Keywords:** Veterans, Suicidal ideation, Suicide attempt, Region, Division, State

## Abstract

**Background:**

Veteran suicide remains a major public health concern; rates increased 64.3% from 2001 to 2022 and substantial geospatial variation exists, with state-level rates ranging from 15.4/100,000 (Maryland) to 87.1/100,000 (Montana). Surveillance of suicidal ideation (SI) and suicide attempts (SA) can provide insights to reduce suicide risk within communities.

**Methods:**

A population-based, cross-sectional survey of 17,949 Veterans residing in all 50 U.S. states, the District of Columbia, Puerto Rico, and U.S. Pacific Island (PI) Territories, was conducted in 2022 to assess SI and SA prevalence. Lifetime and post-military SI and SA and past-year SI prevalence were estimated by Census region, division, and state. Prevalence ratios were calculated for post-military SI and SA to assess differences by division, accounting for demographic covariates (i.e., age, race, gender, rurality, and time since military separation). Methods used in lifetime SA and considered in past-year SI were also examined by region.

**Results:**

The West had the highest prevalence of lifetime (36.94%; 95%CI = 34.65–39.23) and post-military SI (28.73%; 95%CI = 26.51–30.96), significantly higher than all other regions except for PI Territories and Puerto Rico. PI Territories had the highest prevalence of past-year SI (15.68%; 95%CI = 10.91–20.44) and lifetime (9.86%; 95%CI = 6.36–13.37) and post-military SA (5.67%; 95%CI = 3.21–8.14). At the divisional level, the Pacific West (29.12%; 95%CI = 26.01–32.23) and West South Central (29.09%; 95%CI = 26.18-32.00) divisions had the highest prevalence of post-military SI, while West South Central had the highest prevalence of post-military SA (6.89%; 95%CI = 5.07–8.70), and the PI Territories remained highest for lifetime SA. After adjusting for covariates, numerous significant differences across divisions were observed. Differences in suicide methods considered and used were also observed across regions.

**Conclusions:**

Variability in SI and SA prevalence among Veterans at state, divisional and regional levels supports the need for nuanced surveillance efforts, along with targeted prevention efforts in areas at greatest risk.

**Supplementary Information:**

The online version contains supplementary material available at 10.1186/s40621-025-00584-y.

## Introduction

Suicide among U.S. Veterans continues to be a serious public health concern; in 2022, suicide was the 12 th leading cause of death among Veterans, and suicide rates increased 64.3% from 2001 (17.1/100,000)^1^ to 2022 (28.1/100,000) [1, 2]. Further, in 2022, while Veterans made up approximately 6.1% of the U.S. adult population, 13.4% of adult suicides in 2022 occurred among Veterans [2, 3]. However, there is significant geospatial variation in suicide rates among Veterans, which range from 25.0/100,000 in the Northeast to 40.4/100,000 in the West [4]. Identifying geographical differences in Veteran suicide rates can help with tailoring community-based prevention efforts.

Nonetheless, Census regions, which are used to calculate regional suicide rates, are large and heterogenous. This may obscure more granular geographic differences in rates. Indeed, state-level suicide rates among Veterans in 2022 ranged from 15.4/100,000 (Maryland) to 87.1/100,000 (Montana) [4]. Notably, both Montana and California (29.1/100,000) are in the West region, but have vastly different suicide rates among Veterans; this may be due to differences in rurality, access to firearms, and/or socioeconomic factors [5, 6].

Although division and state-level estimates provide a clearer picture of geographic trends in Veteran suicide rates, such findings are often limited. As part of the annual suicide prevention report, the Department of Veterans Affairs (VA) publishes state data sheets [4]. While helpful in providing states with information specific to their Veteran population, state suicide rates are limited to crude, rather than age- and sex-adjusted, rates, limiting comparisons between states [4]. Furthermore, suicide rates for some territories are not available, even unadjusted. Estimates at the Census division level are not available, nor can they be calculated from the VA data appendix due to suppression of values where the number of suicide deaths is less than 10 to protect privacy. Thus, additional approaches to understand Veterans’ state- and division-level suicide risk are necessary.

Surveillance of suicidal ideation (SI) and suicide attempts (SA) can provide valuable insight into geographical trends in suicide risk that can inform prevention. SI and SA are among the strongest predictors of future suicidal behavior, including suicide [7]. Specifically, Veterans who attempt suicide have nearly twice the hazard of all-cause mortality than Veterans who do not after adjustment for age and gender [8]. Moreover, SI and SA can be distressing to those who experience them and have additional physical and psychosocial impacts; Veterans with a history of SA have worse subsequent psychological well-being compared to those with no history of SA even after accounting for mental health symptoms (e.g., depression) [9]. In the general U.S. population, non-fatal self-directed violence, including SI and SA, costs an average of $13.1 billion in medical costs and $3.2 billion in work loss each year [10]. Furthermore, suicide, SA, and SI among Veterans have significant psychological impacts for family members, friends, and fellow Veterans, including SI and SA, mental health symptoms, and social isolation [11]. Thus, SI and SA are important clinical outcomes. Understanding how they vary across regions, divisions, and states can help tailor efforts intended to improve well-being and prevent Veteran suicide.

While studies have examined the prevalence of SI and SA among Veterans, none have obtained a large, nationally representative sample equipped to examine these geographical differences. To address this gap, the current manuscript aimed to describe geographic variation in prevalence of SI and SA among Veterans, using data from the *Assessing Social and Community Environments with National Data for Veteran Suicide Prevention* (ASCEND) study, a population-based, cross-sectional national survey designed to conduct non-fatal suicidal self-directed violence surveillance [12]. ASCEND data were analyzed to describe and visualize the geographic distribution of SI and SA based on region, division, and state/territory, as well as to describe suicide methods considered and used by region. We also compared the prevalence of SI and SA across Census divisions.

## Methods

### Sample

ASCEND is a recurring, population-based, cross-sectional survey. Wave 1 (2022) included a main study sample (all U.S. states, Washington D.C., and Puerto Rico); and a U.S. Pacific Islands (PI) Territories pilot sample (Guam, American Samoa, and the Commonwealth of the Northern Mariana Islands [CNMI]); both samples are included in the present manuscript [12, 13].^2^ Both samples were drawn from a population frame of all living U.S. Veterans (*N* = 16,738,616, as of 06/2021), constructed from U.S. Veterans Eligibility Trends and Statistics (USVETS) and the VA-DoD Identity Repository (VADIR) data [14]. Frame data were used to classify records according to groups pertinent to the stratified sampling design (i.e., state/territory of residence, sex, race/ethnicity, and date of separation for main sample and territory of residence for PI sample). Additional sampling details have been described previously [12, 13]. A total of 17,949 Veterans participated in Wave 1 (17,396 in the main study; 553 in the PI pilot). A 10-week recruitment protocol was implemented from 03/2022 to 06/2022; across both the main and pilot samples, respondents completed the survey by web (74.5%), paper (25.1%), or phone (0.5%) [12]. Response rates for the main and pilot samples were 19.2% and 21.6%, respectively. Yield by state/territory is provided in Supplemental Table 1.

### Measures

The Wave 1 survey assessed a broad range of constructs, including demographic and military characteristics, SI and SA, and risk and protective factors for suicide. Constructs relevant to the present analyses are described.

#### SI and SA

A modified self-report version of the Columbia Suicide Severity Rating Scale (C-SSRS) was administered [12, 15]. The C-SSRS is a widely used measure of non-fatal suicidal self-directed violence, and is used nationally in VHA to screen for suicide risk. The ASCEND version was expanded and developed in a pilot study in which feedback from Veterans was received [16]. Lifetime SI was defined as a positive response to any questions regarding SI (i.e., thoughts of killing self), SI with consideration of method (i.e., thoughts about how you might kill yourself), and/or SI with a specific plan. SA was defined as having ever purposely hurt oneself with at least some intent to die. Respondents who endorsed experiencing lifetime SI or SA were asked about timing of SI and SA relative to their military service (before, during, and after) and about SI within the past year. Those who endorsed past-year SI were asked about methods considered during that time. Respondents who endorsed lifetime SA were also asked about method(s) used in prior attempts.

#### Geographical variables

The state or territory of residence for each participant was obtained from contact information provided on completed surveys. Regions (Northeast, Midwest, South, West)^3^ and divisions (New England, Mid-Atlantic, East North Central, West North Central, South Atlantic, East South Central, West South Central, Mountain West, Pacific West)^4^ were classified using standard US Census Bureau definitions [17]. PI Territories included Guam, CNMI, and American Samoa. While Puerto Rico is in the Caribbean Island Territories region, it was the only Caribbean Island territory included in Wave 1 and thus was analyzed separately as both a region and division for this analysis. Rurality was classified based on the rural-urban commuting area (RUCA) code of the zip code provided from contact information on completed surveys^5^. Veterans with an invalid zip code (*n* = 39) or a zip code that corresponded with an Army Post Office (APO) or Fleet Post Office (FPO) (*n* = 8) were coded as missing for rurality.

#### Demographics and military service characteristics

Self-reported demographics included: age, gender, and race. Time since last military separation was calculated based on year of last separation (self-reported) and year of survey completion.

### Statistical analysis

All analyses were conducted using SAS version 9.4 and R version 4.3.2. and weighted to account for the complex survey design and propensity for non-response to reduce bias and enhance generalizability of results [12]. Non-response weights adjusted for differential non-response among sampled groups. Specifically, response propensity was associated with age, race, ethnicity, rurality, recent use of VHA services, and time since military separation. Thus, non-response weights accounted for potential bias introduced by these associations [12]. In addition to non-response adjustment, final weights used in all analyses included design weights and eligibility adjustment, and were raked to align with the Veteran population. Unweighted and weighted frequencies, proportions and 95% confidence intervals (CIs) were calculated for lifetime and post-military SI and SA, as well as past-year SI, by region, division, and state/territory. Similarly, proportions and 95% CIs were calculated for methods considered among those with past-year SI and methods used among those with lifetime SA, by region. All prevalence estimates were evaluated using the National Center for Health Statistics Data Presentation Standards for Proportions [18]. Estimates deemed unreliable based on these standards were suppressed; for this reason, it was not possible to report methods considered or used by division or state/territory. Reliable estimates were compared based upon evaluation of overlapping CIs; when 95% CI did not overlap, estimates were considered significantly different. Prevalence ratios (PRs) were calculated using weighted modified Poisson regression models with robust standard errors to compare post-military and past-year SI and post-military SA by division, accounting for covariates of interest [19]. Covariates in adjusted models, selected a priori based on literature on demographic differences in suicide rates, included age (18–34, 35–49, 50–64, 65+), race (White, Black, American Indian/Alaska Native, Asian, Native Hawaiian, Pacific Islander, other, multiracial), gender (man, woman, transgender, non-binary/other), rurality (rural or urban), and time since military separation (< 4 years, 4–9 years, 10 + years) [2]. No specific division was used as a reference group for comparison; all pairwise comparisons were made. PI Territories were excluded from modeling due to differences in sampling design and subsequent weight construction precluding their inclusion in the main sample; Puerto Rico was excluded from modeling due to small sample size.

## Results

### Population characteristics

Within the main sample, Veterans primarily lived in the South (44.06%; 95%CI = 43.53–44.60), followed by the West (22.21%; 95%CI = 21.70–22.72), Midwest (20.52%; 95%CI = 20.08–20.95), Northeast (12.82%; 95%CI = 12.48–13.61), and Puerto Rico (0.39%; 95%CI = 0.31–0.47). By state/territory, the highest proportion of Veterans lived in Texas (South; 8.52%; 95%CI = 8.23–8.81), followed by California (West; 7.94%; 95%CI = 7.54–8.33) and Florida (South; 7.63%; 95%CI = 7.38–7.88) (Supplemental Table 2). Within the PI Territories sample, most Veterans lived in Guam (86.84%; 95%CI = 82.73–90.94) (Supplemental Table 2).

The age distribution of Veterans did not differ significantly by region, with the largest portion of Veterans > 65 years (range: 40.41–43.55%) and the smallest portion of Veterans 18–34 years (range: 8.83–10.91%) across regions (Supplemental Table 3). Approximately 90% of Veterans were men across all regions, though a larger proportion of women Veterans lived in the South (12.17% 95%CI = 11.71–12.63) relative to all other regions. While the majority of Veterans were White, there were racial differences across regions. The South had a significantly higher proportion of Black Veterans (16.09%; 95%CI = 15.17–17.02), and the PI Territories (9.94%; 95%CI = 6.69, 13.19; Supplemental Table 3) and West (3.32%; 95%CI = 2.67–3.97) had a higher proportion of Asian Veterans, compared to other regions. Additionally, the PI Territories had a higher proportion of Pacific Islander (45.87%; 95%CI = 37.29–54.46) and multi-racial (23.98%; 95%CI = 10.83–37.12) Veterans. Nearly one-third of Veterans in the Midwest (31.26%; 95%CI = 29.24–33.28) lived in a rural area, more than all other regions. Finally, approximately 90% of Veterans in all regions had been separated from military service for 10 years or more.

In the overall main sample (i.e., all 50 states, Washington, D.C., and Puerto Rico), the prevalence of lifetime SI was 31.98% (95%CI = 30.97–32.99), prevalence of SI after military separation was 25.88% (95%CI = 24.91–26.85), and prevalence of past-year SI was 12.69% (95%CI = 11.90–13.47) [12]. For SA, the prevalence of lifetime SA in the main sample was 6.99% 95%CI = 6.41–7.56) and SA after military separation was 4.88% (95%CI = 4.39–5.36) [12].

### Regional estimates

#### SI

**Fig. 1:**
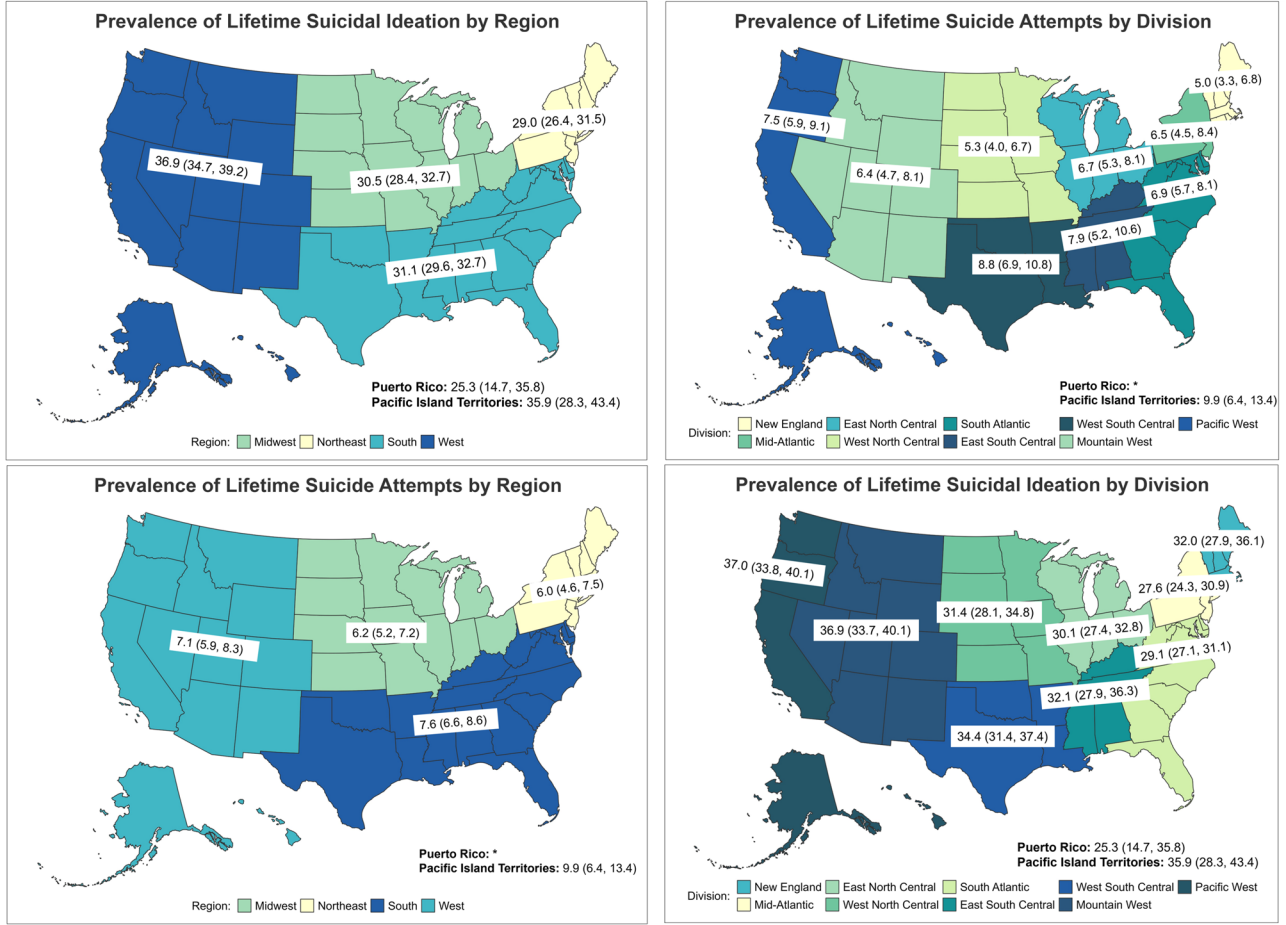
Prevalence of Lifetime Suicidal Ideation and Suicide Attempts by Region and Division

In the West, 36.94% (95%CI = 34.65–39.23) of Veterans reported lifetime SI, which was significantly higher than in the South (31.11%; 95%CI = 29.56–32.66), Midwest (30.54%; 95%CI = 28.42–32.65), and Northeast (28.95%; 95%CI = 26.36–31.53) (Table 1). PI Territories had a similarly high prevalence of lifetime SI (35.86%; 95%CI = 28.34–43.39) to the West. Puerto Rico had the lowest regional prevalence of lifetime SI (25.28%; 95%CI = 14.72–35.84) (Fig. 1).

**Table 1:** Prevalence estimates of lifetime and post-military suicidal ideation and suicide attempts and past-year suicidal ideation by census region

	Northeast			Midwest			South		
	Unweighted	Weighted		Unweighted	Weighted		Unweighted	Weighted	
	N	N	% (95% CI)	N	N	% (95% CI)	N	N	% (95% CI)
**Suicidal Ideation**									
Lifetime	667	575,243	28.95 (26.36, 31.53)	1,020	973,931	30.54 (28.42, 32.65)	1,719	2,123,368	31.11 (29.56, 32.66)
Post-Military	517	449,190	22.76 (20.34, 25.18)	821	795,465	25.08 (23.06, 27.11)	1,386	1,754,065	25.77 (24.28, 27.26)
Past Year	220	193,826	9.69 (7.96, 11.43)	372	374,306	11.69 (10.15, 13.23)	659	936,267	13.62 (12.35, 14.89)
**Suicide Attempts**									
Lifetime	153	119,449	6.02 (4.58, 7.47)	248	197,385	6.20 (5.18, 7.23)	412	517,345	7.60 (6.63, 8.57)
Post-Military	110	95,284	4.77 (3.47, 6.07)	175	142,027	4.46 (3.55, 5.36)	290	374,896	5.47 (4.62, 6.32)
	**West**			**Pacific Island Territories**			**Puerto Rico**		
**Suicidal Ideation**									
Lifetime	1,363	1,267,225	36.94 (34.65, 39.23)	172	3,403	35.86 (28.34, 43.39)	54	15,185	25.28 (14.72, 35.84)
Post-Military	1,064	986,505	28.73(26.51, 30.96)	126	2,394	25.34 (19.29, 31.39)	43	11,948	20.13 (10.17, 30.08)
Past Year	476	469,438	13.55 (11.81, 15.29)	71	1,489	15.68 (10.91, 20.44)	*		
**Suicide Attempts**									
Lifetime	278	242,242	7.07 (5.89, 8.25)	50	934	9.86 (6.36, 13.37)	*		
Post-Military	179	143,049	4.15 (3.29, 5.00)	31	533	5.67 (3.21, 8.14)	*		

* Denotes suppressed due to unreliable estimate

**Fig. 2:**
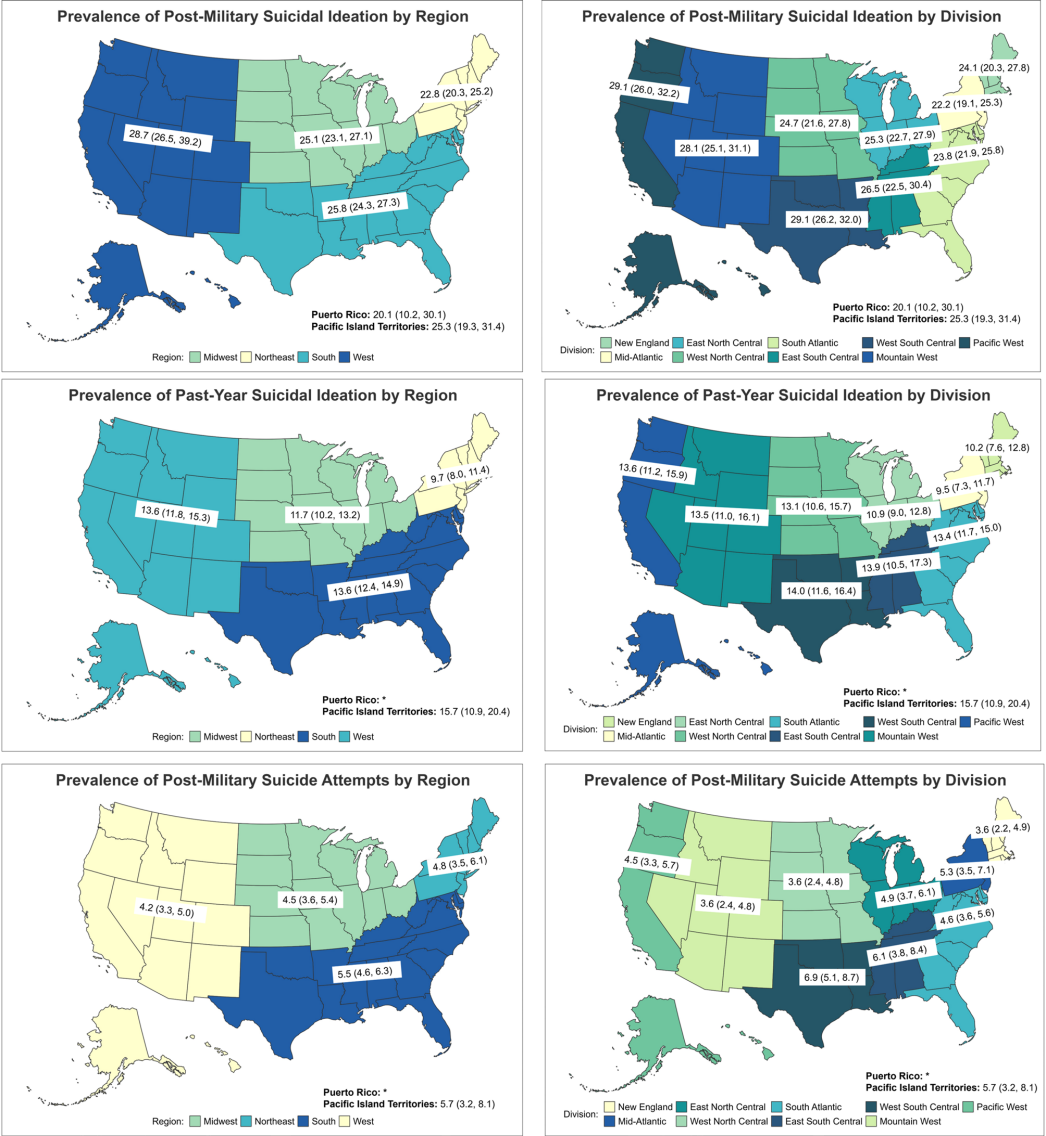
Maps of Prevalence Estimates of Post-Military and Past-Year Suicidal Ideation and Post-Military Suicide Attempt by Region and Division

SI after separation from military service was also highest in the West (28.73%; 95%CI = 26.51–30.96), followed by the South (25.77%; 95%CI = 24.28–27.26), PI Territories (25.34%; 95%CI = 19.29–31.39), Midwest (25.08%; 95%CI = 23.06–27.11), Northeast (22.76%; 95%CI = 20.34–25.18), and Puerto Rico (20.13%; 95%CI = 10.17–30.08). Post-military SI prevalence in the West was significantly higher than in the Northeast (Fig. 2).

Veterans in PI Territories (15.68%; 95%CI = 10.91–20.44) had the highest prevalence of past-year SI, followed by those in the West (13.55%; 95%CI = 11.81–15.29), and South (13.62%; 95%CI = 12.35–14.89). Past-year SI prevalence was lowest in the Northeast (9.69%; 95%CI = 7.96–11.43) (Fig. 2).

Among Veterans who reported suicidal ideation in the past year, gunshot was the most commonly considered method in the Midwest (43.65%; 95%CI = 36.43–50.86), West (40.92%; 95%CI = 33.58–48.27), and South (39.01%; 95%CI = 33.81–44.20), while motor vehicle crash was the most commonly considered method in the Northeast (35.68%; 95%CI = 26.38–44.97) and PI Territories (32.77%; 95%CI = 19.38–46.16) (Table 2). Overdose of medications was among the three most commonly considered methods in all regions; however, overdose of illegal drugs was the fourth most commonly considered method in the Midwest (11.32%; 95%CI = 6.43–16.21), but was not among the five most common methods in any other region.

**Table 2:** Suicidal ideation methods considered, by region

	Northeast			Midwest			South		
	Unweighted	Weighted		Unweighted	Weighted		Unweighted	Weighted	
	N	N	% (95% CI)	N	N	% (95% CI)	N	N	% (95% CI)
**SI Methods Considered**									
Gunshot	67	63,208	32.61 (23.79, 41.44)	130	163,180	43.65 (36.43, 50.86)	223	363,134	39.01 (33.81, 44.20)
Motor Vehicle Crash	74	69,148	35.68 (26.38, 44.97)	113	114,102	30.52 (23.86, 37.17)	210	333,025	35.77 (30.61, 40.93)
Overdose of Medications	80	63,609	32.82 (24.06, 41.58)	119	102,393	27.39 (21.32, 33.45)	226	292,025	31.37 (26.60, 36.14)
Jumping from a High Place	*			23	28,417	7.60 (3.70, 11.50)	53	89,461	9.61 (5.92, 13.30)
Suffocation or Asphyxiation	17	19,728	10.18 (3.66, 16.70)	34	29,543	7.90 (4.46, 11.35)	46	99,130	10.65 (6.62, 14.68)
Hanging	22	22,200	11.45 (5.46, 17.45)	27	39,571	10.58 (5.53, 16.63)	49	83,834	9.00 (5.75, 12.26)
Cutting or Stabbing	17	14,620	7.54 (2.91, 12.17)	28	28,716	7.68 (3.63, 11.74)	49	89,099	9.57 (5.15, 12.99)
Overdose of Illegal Drugs	23	16,882	8.71 (4.51, 12.91)	30	42,314	11.32 (6.43, 16.21)	46	68,017	7.31 (4.84, 9.77)
Drowning	*			21	22,311	5.97 (2.83, 9.11)	30	48,712	5.23 (2.60, 7.86)
Any Other Method	*			23	12,763	3.41 (1.52, 5.31)	22	29,681	3.19 (1.31, 5.07)
	**West**			**Pacific Island Territories**			**Puerto Rico**		
**SI Methods Considered**									
Gunshot	170	191,066	40.92 (33.58, 48.27)	20	362	24.30 (11.98, 36.62)	*		
Motor Vehicle Crash	149	178,829	38.30 (30.75, 45.85)	22	488	32.77 (19.38, 46.16)	*		
Overdose of Medications	153	136,035	29.14 (21.89, 36.38)	22	362	24.31 (13.48, 35.14)	*		
Jumping from a High Place	52	78.381	16.79 (9.56, 24.02)	15	271	18.21 (8.30, 28.12)	*		
Suffocation or Asphyxiation	46	61,323	13.13 (7.83, 18.43)	*			*		
Hanging	52	56,397	12.08 (8.06, 16.10)	*			*		
Cutting or Stabbing	43	53,097	11.37 (6.72, 16.03)	*			*		
Overdose of Illegal Drugs	46	49,342	10.57 (5.68, 15.46)	*			*		
Drowning	22	27,852	5.97 (3.04, 8.89)	*			*		
Any Other Method	*			*			*		

* Denotes suppressed due to unreliable estimate

#### SA

Prevalence estimates for Puerto Rico were suppressed due to potential unreliability (Table 1). The highest proportion of lifetime SA was observed for Veterans in PI Territories (9.86%; 95%CI = 6.36–13.37), followed by the South (7.60%; 95%CI = 6.63–8.57), West (7.07%; 95%CI = 5.89–8.25), Midwest (6.20%; 95%CI = 5.18–7.23), and Northeast (6.02%; 95%CI = 4.58–7.47), though differences were not significant (Fig. 1). Similarly, Veterans in PI Territories and the South appeared to have a higher prevalence of SA following separation from the military (range: 5.67–5.47%) while prevalence was lower among Veterans in the West, Midwest and Northeast (range: 4.15–4.77%), though these differences were also not statistically significant (Fig. 2).

Among Veterans with a lifetime suicide attempt, the most common method used was overdose of medications, followed by cutting/stabbing and gunshot across all regions (Table 3). Nearly two-thirds of Veterans in the Midwest (61.59%; 95%CI = 52.91–70.26) and South (61.54%; 95%CI = 54.67–68.40) reported using overdose of medications as a suicide attempt method, while approximately half of Veterans in the Northeast (49.52%; 95%CI = 36.66–62.38) and West (52.13%; 95%CI = 43.33–60.94) did. Of note, more Veterans in the Northeast reported using gunshot as a suicide attempt method (23.07%; 95%CI = 10.28–35.86) than in any other region, although this difference was not statistically significant.

**Table 3:** Suicide attempt methods used among veterans with a lifetime suicide attempt, by region

	Northeast			Midwest			South		
	Unweighted	Weighted		Unweighted	Weighted		Unweighted	Weighted	
	N	N	% (95% CI)	N	N	% (95% CI)	N	N	% (95% CI)
**SA Methods Used**									
Overdose of Medication	78	56,763	49.52 (36.66, 62.38)	152	114,933	61.59 (52.91, 70.26)	241	305,402	61.54 (54.67, 68.40)
Cutting or Stabbing	41	29,643	25.86 (15.18, 36.54)	78	57,910	31.03 (22.30, 39.76)	98	132,675	26.73 (20.04, 33.42)
Gunshot	16	26,444	23.07 (10.28, 35.86)	34	32,444	17.38 (10.70, 24.07)	59	94,713	19.08 (13.47, 24.70)
Motor Vehicle Crash	21	25,095	21.89 (10.16, 33.63)	36	29,141	15.62 (9.36, 21.87)	65	92,010	18.54 (13.32, 23.76)
Hanging	*			26	30,891	16.49 (8.70, 24.28)	38	68,614	13.73 (8.54, 18.91)
Suffocation or Asphyxiation	*			19	19,207	10.29 (3.65, 16.93)	27	48,761	9.82 (4.74, 14.91)
Overdose of Illegal Drugs	*			*			41	51,786	10.43 (6.51, 14.36)
Jumping from a High Place	*			*			31	48,651	9.78 (4.75, 14.82)
Drowning	*			*			18	25,366	5.11 (2.18, 8.05)
Any Other Method	20	18,287	15.95 (6.04, 25.87)	24	14,640	7.84 (3.63, 12.06)	50	67,136	13.53 (9.08, 17.98)
	**West**			**Pacific Island Territories**			**Puerto Rico**		
**SA Methods Used**									
Overdose of Medication	154	118,847	52.13 (43.33, 60.94)	*			*		
Cutting or Stabbing	71	63,312	27.77 (19.96, 35.58)	*			*		
Gunshot	51	47,161	20.69 (13.18, 28.19)	*			*		
Motor Vehicle Crash	45	39,129	17.16 (11.03, 23.30)	*			*		
Hanging	29	31,559	13.61 (7.92, 19.30)	*			*		
Suffocation or Asphyxiation	24	32,630	14.31 (7.07, 21.56)	*			*		
Overdose of Illegal Drugs	21	11,716	5.14 (2.11, 8.17)	*			*		
Jumping from a High Place	17	15,856	6.96 (3.05, 10.86)	*			*		
Drowning	*			*			*		
Any Other Method	28	25,022	10.98 (5.85, 16.10)	*			*		

* Denotes suppressed due to unreliable estimate

### Divisional estimates

#### SI

The Pacific West (36.99%; 95%CI = 33.83–40.07) and Mountain West (36.86%; 95%CI = 33.65–40.07) had the highest prevalence of lifetime SI (Fig. 1). These two divisions had significantly higher prevalence than the Mid-Atlantic (27.59%; 95%CI = 24.33–30.86), South Atlantic (29.11%; 95%CI = 27.11–31.10) and East North Central (30.07%; 95% CI = 27.36–32.78). West South Central (34.41%; 95% CI = 31.43–37.40) had higher prevalence than the Mid-Atlantic and South Atlantic (Table 4). There were no other significant differences.

**Table 4:** Prevalence estimates of lifetime and post-military suicidal ideation and suicide attempts and past-year suicidal ideation by census division

	Division 1: New England			Division 2: Mid-Atlantic			Division 3: East North Central		
	Unweighted	Weighted		Unweighted	Weighted		Unweighted	Weighted	
	N	N	% (95% CI)	N	N	% (95% CI)	N	N	% (95% CI)
**Suicidal Ideation**									
Lifetime	368	197,120	31.95 (27.85, 36.05)	299	378,123	27.59 (24.33, 30.86)	524	625,830	30.07 (27.36, 32.78)
Post-Military	283	147,418	24.05 (20.33, 27.77)	234	301,772	22.18 (19.09, 25.27)	428	522,846	25.28 (22.65, 27.90)
Past Year	119	63,040	10.18 (7.55, 12.82)	101	130,786	9.47 (7.26, 11.69)	178	228,149	10.91 (8.99, 12.84)
**Suicide Attempts**									
Lifetime	81	30,990	5.03 (3.27, 6.80)	72	88,459	6.47 (4.54, 8.41)	130	138,565	6.67 (5.28, 8.07)
Post-Military	54	22,085	3.57 (2.22, 4.91)	56	73,199	5.31 (3.53, 7.09)	96	102,030	4.91 (3.68, 6.13)
	**Division 4: West North Central**			**Division 5: South Atlantic**			**Division 6: East South Central**		
**Suicidal Ideation**									
Lifetime	496	348,101	31.41 (28.08, 34.75)	994	1,099,701	29.11 (27.11, 31.10)	253	345,390	32.06 (27.87, 36.26)
Post-Military	393	272,619	24.72 (21.61, 27.83)	792	899,508	23.84 (21.92, 25.76)	207	282,488	26.46 (22.51, 30.41)
Past Year	194	146,158	13.14 (10.59, 15.70)	394	508,455	13.35 (11.69, 15.01)	95	150,841	13.90 (10.46, 17.34)
**Suicide Attempts**									
Lifetime	118	58,821	5.31 (3.95, 6.68)	233	258,407	6.86 (5.67, 8.06)	62	84,951	7.90 (5.20, 10.61)
Post-Military	79	39,997	3.61 (2.41, 4.81)	158	173,003	4.56 (3.55, 5.56)	48	66,183	6.09 (3.75, 8.44)
	**Division 7: West South Central**			**Division 8: Mountain West**			**Division 9: Pacific West**		
**Suicidal Ideation**									
Lifetime	472	678,276	34.41 (31.43, 37.40)	685	506,259	36.86 (33.65, 40.07)	678	760,966	36.99 (33.83, 40.07)
Post-Military	387	572,070	29.09 (26.18, 32.00)	541	382,604	28.14 (25.14, 31.14)	523	603,901	29.12 (26.01, 32.23)
Past Year	170	276,971	14.00 (11.59, 16.40)	235	185,898	13.52 (10.98, 16.06)	241	283,541	13.57 (11.22, 15.93)
**Suicide Attempts**									
Lifetime	117	173,986	8.84 (6.86, 10.82)	140	87,935	6.41 (4.71, 8.11)	138	154,307	7.51 (5.90, 9.12)
Post-Military	84	135,710	6.89 (5.07, 8.70)	91	48,986	3.58 (2.38, 4.77)	88	94,063	4.52 (3.34, 5.70)

Regarding SI following separation from military service, the Pacific West (29.12%; 95%CI = 26.01–32.23), and West South Central divisions (29.09%; 95%CI = 26.18–32.00) had the highest prevalence, significantly higher than Mid-Atlantic (22.18%; 95%CI = 19.09–25.27) or South Atlantic (23.84%; 95%CI = 21.92–25.76) divisions (Fig. 2). No other divisions differed from each other. No significant differences were observed in past-year SI by division (Fig. 2), although prevalence estimates appear somewhat lower for East North Central (10.91%; 95%CI = 8.99–12.84), New England (10.18%, 95%CI = 7.55–12.82), and Mid-Atlantic (9.47%; 95%CI = 7.26–11.69) than in other divisions (range: 13.14–15.68%).

#### SA

PI Territories (9.86%; 95%CI = 6.36–13.37) and West South Central (8.84%; 95%CI = 6.86–10.82) had the highest prevalence of lifetime SA (Fig. 1). Prevalence was significantly higher in West South Central than the West North Central (5.31%; 95%CI = 3.95–6.68) and New England (5.03%; 95%CI = 3.27–6.80) divisions (Table 4).

West South Central (6.89%; 95%CI = 5.07–8.70), East South Central (6.09%; 95%CI = 3.75–8.44) and PI Territories (5.67%; 95%CI = 3.21–8.14) had the highest prevalence of SA after separation from the military (Fig. 2). The West North Central (3.61%; 95%CI = 2.41–4.81) and Mountain West (3.58%; 95%CI = 2.38–4.77) divisions had significantly lower prevalence compared to the West South Central division, but there were no other significant differences.

### State/territory-level estimates

#### SI

**Fig. 3:**
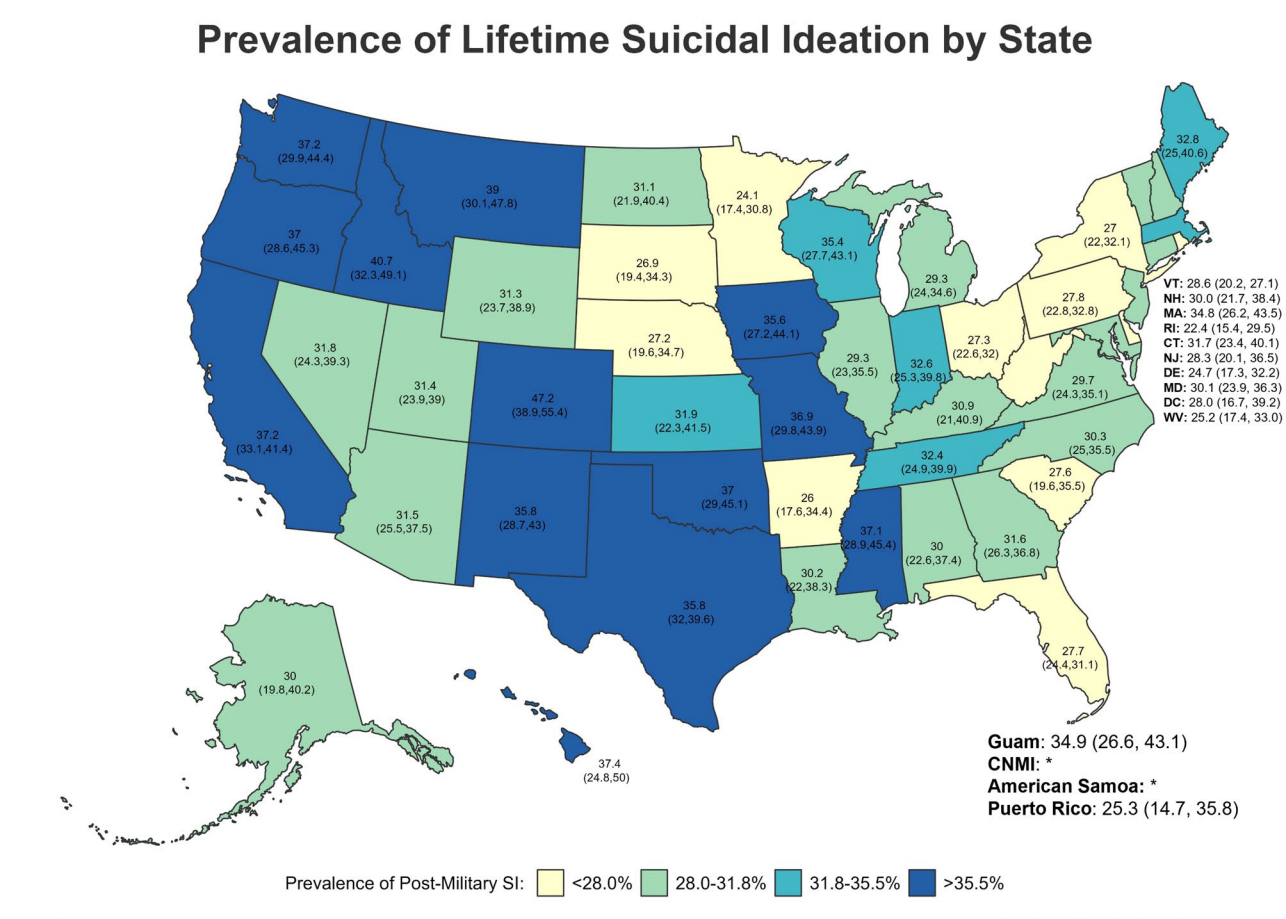
Prevalence of Lifetime Suicidal Ideation by State

**Fig. 4:**
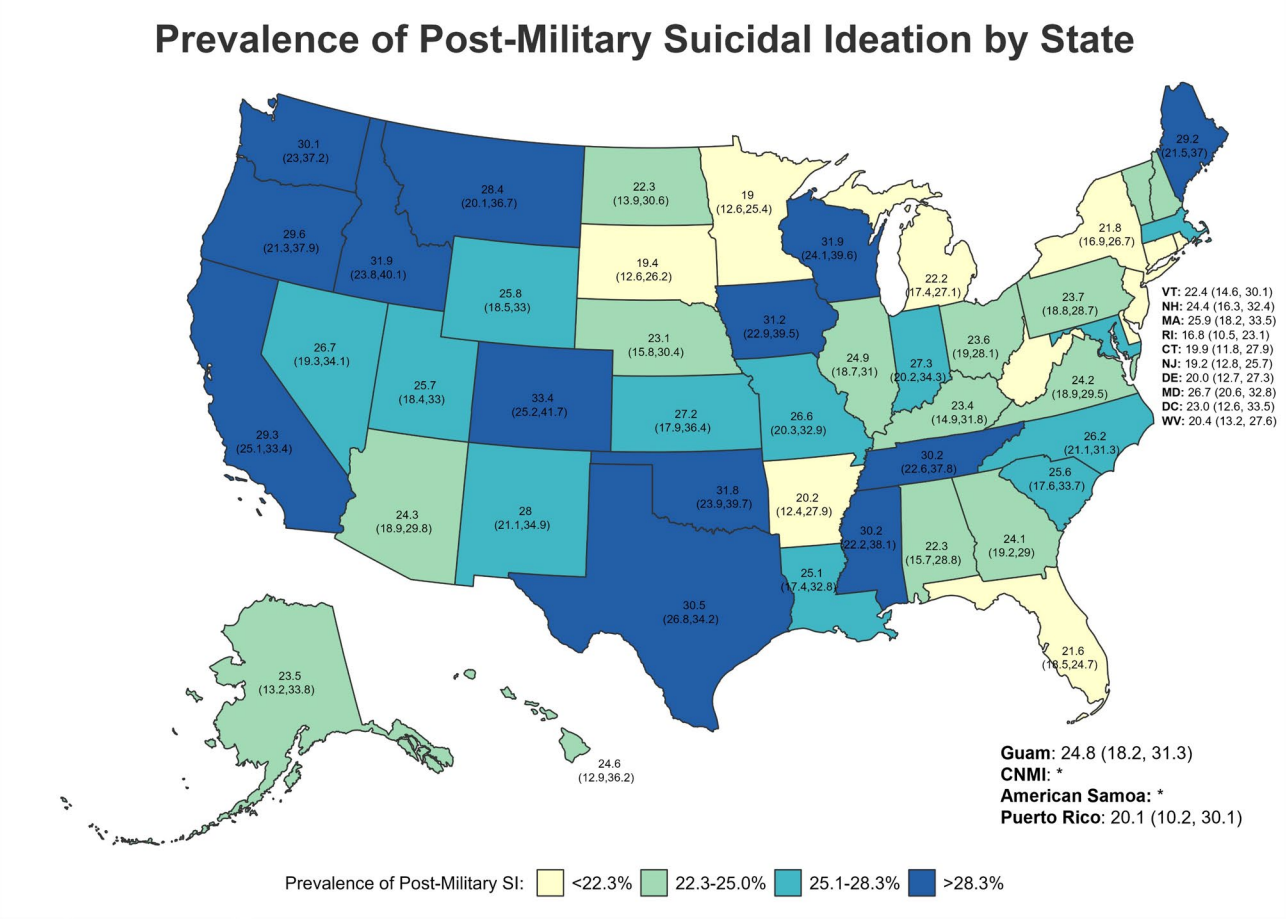
Prevalence of Post-Military Suicidal Ideation by State

**Fig. 5:**
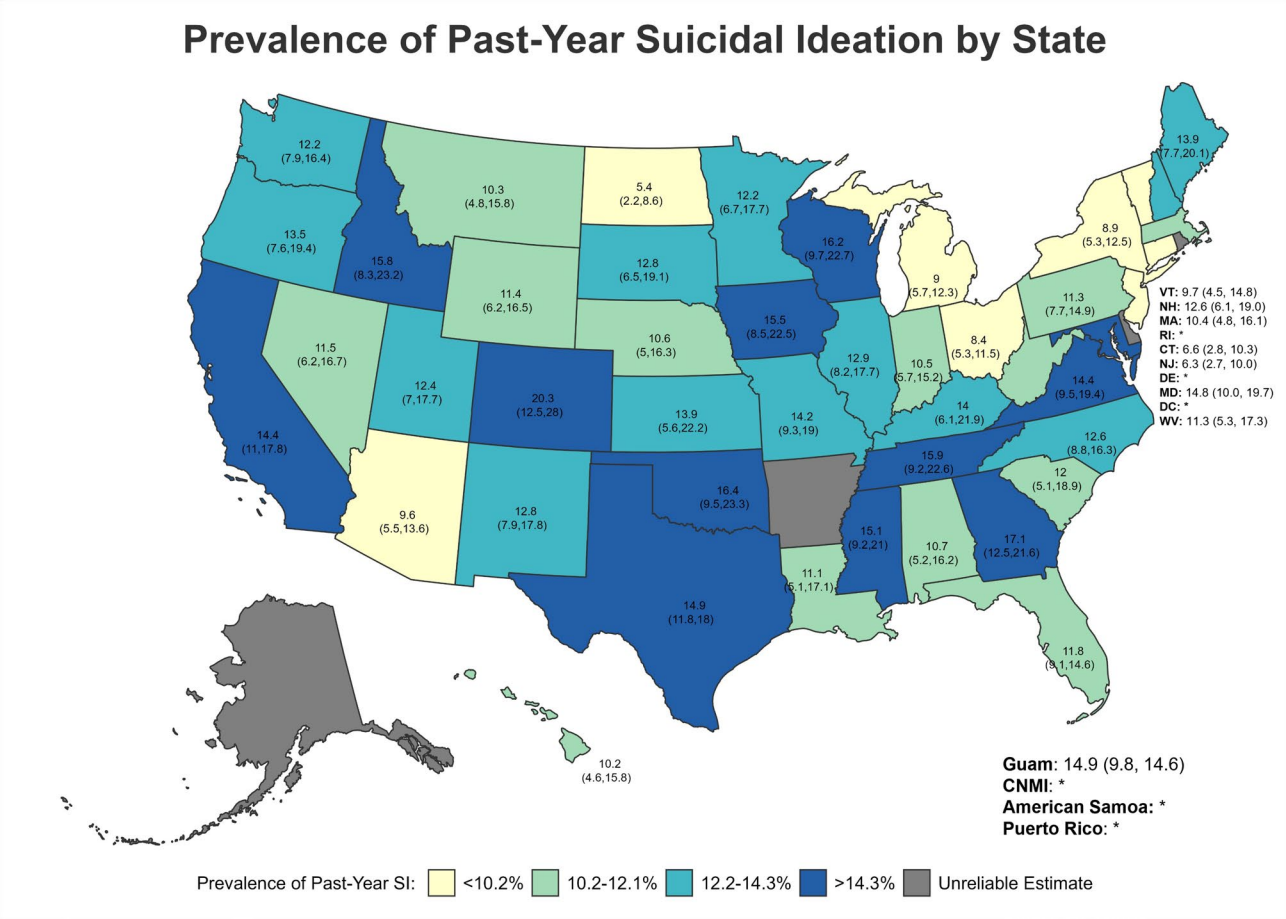
Prevalence of Past-Year Suicidal Ideation by State

Across states/territories, the five with the highest lifetime SI prevalence (Fig. 3) were all in the West—specifically, Colorado (47.18%; 95%CI = 38.93–55.44), Idaho (40.74%; 95%CI = 32.34–49.14), Montana (38.95%; 95%CI = 30.13–47.78), Hawaii (37.43%; CI = 24.83–50.03), and California (37.23%; 95%CI = 33.06–41.40) (Table 5; Figs. 3, 4 and 5). However, for post-military SI (Fig. 4), the five highest prevalence estimates were in Colorado (33.43%; 95%CI = 25.16–41.71; West), Idaho (31.94%; 95%CI = 23.82–40.06; West), Wisconsin (31.86%; 95%CI = 24.14–39.59; Midwest), Oklahoma (31.77%; 95%CI = 23.88–39.66; South), and Iowa (31.20%; 95%CI = 22.89–39.52; Midwest). Finally, Colorado (20.27%; 95%CI = 12.51–28.03; West), Georgia (17.06%; 95%CI = 12.49–21.63; South), Oklahoma (16.41%; 95%CI = 9.47–23.34; South), Wisconsin (16.18%; 95%CI = 9.66–22.71; Midwest), and Tennessee (15.91; 95%CI = 6.21–22.61; South) had the highest prevalence of past-year SI (Fig. 5). Estimates of past-year SI prevalence were suppressed in some states and territories due to unreliable estimates.

**Table 5:** Prevalence estimates of lifetime, post-military, and past-year suicidal ideation, by state/territory

	Lifetime			Post-Military			Past-Year		
	Unweighted	Weighted		Unweighted	Weighted		Unweighted	Weighted	
	N	N	% (95% CI)	N	N	% (95% CI)	N	N	% (95% CI)
Alaska	56	17,664	30.03 (19.83, 40.22)	38	13,736	23.52 (13.24, 33.79)	*		
Alabama	70	91,255	29.98 (22.55, 37.40)	54	66,774	22.28 (15.74, 28.81)	22	32,766	10.7 (5.17, 16.24)
Arkansas	39	46,101	26.03 (17.63, 34.44)	31	35,812	20.19 (12.44, 27.93)	*		
American Samoa	*			*			*		
Arizona	99	127,054	31.5 (25.48, 37.52)	77	97,789	24.34 (18.90, 29.77)	28	38,653	9.58 (5.54, 13.62)
California	358	454,094	37.23 (33.06, 41.40)	279	359,623	29.26 (25.14, 33.37)	128	178,249	14.4 (11.00, 17.79)
Colorado	96	157,662	47.18 (38.93, 55.44)	78	109,660	33.43 (25.16, 41.71)	40	67,742	20.27 (12.51, 28.03)
Connecticut	57	40,713	31.73 (23.41, 40.05)	37	25,412	19.89 (11.84, 27.94)	17	8,418	6.57 (2.84, 10.31)
District of Columbia	61	5,458	27.96 (16.73, 39.18)	49	4,464	23.04 (12.59, 33.49)	24	2,360	12.09 (4.79, 19.40)
Delaware	58	13,146	24.74 (17.28, 32.20)	45	10,535	19.97 (12.68, 27.27)	*		
Florida	274	326,567	27.74 (24.39, 31.09)	210	255,316	21.61 (18.49, 24.73)	101	140,716	11.83 (9.10, 14.55)
Georgia	136	185,642	31.56 (26.33, 36.79)	103	141,204	24.06 (19.18, 28.95)	63	101,164	17.06 (12.49, 21.63)
Guam	146	2,870	34.85 (26.61, 43.09)	109	2,029	24.75 (18.19, 31.31)	57	1,227	14.88 (9.81, 19.60)
Hawaii	59	34,054	37.43 (24.83, 50.03)	46	22,304	24.55 (12.93, 36.18)	23	9,385	10.24 (4.63, 15.85)
Iowa	76	56,001	35.61 (27.15, 44.07)	66	48,851	31.2 (22.89, 39.52)	36	24,549	15.54 (8.53, 22.54)
Idaho	94	43,655	40.74 (32.34, 49.14)	71	34,103	31.94 (23.82, 40.06)	29	16,995	15.77 (8.31, 23.22)
Illinois	99	137,436	29.27 (23.00, 35.54)	82	115,850	24.86 (18.74, 30.99)	41	60,983	12.94 (8.20, 17.68)
Indiana	84	106,432	32.56 (25.35, 39.78)	69	87,170	27.26 (20.19, 34.33)	27	34,327	10.48 (5.74, 15.22)
Kansas	65	49,342	31.91 (22.32, 41.51)	56	41,990	27.16 (17.92, 36.39)	24	21,535	13.95 (5.65, 22.25)
Kentucky	51	73,364	30.94 (20.96, 40.93)	43	54,989	23.36 (14.91, 31.81)	21	33,171	13.99 (6.10, 21.89)
Louisiana	49	68,915	30.18 (22.03, 38.33)	40	57,019	25.12 (17.41, 32.84)	17	25,572	11.14 (5.14, 17.13)
Massachusetts	62	80,035	34.83 (26.15, 43.52)	49	59,173	25.86 (18.24, 33.49)	16	24,089	10.44 (4.79, 16.09)
Maryland	87	90,012	30.11 (23.94, 36.28)	74	79,165	26.67 (20.57, 32.77)	41	44,352	14.84 (10.03, 19.65)
Maine	76	30,926	32.81 (25.00, 40.63)	66	26,929	29.24 (21.49, 26.99)	30	13,059	13.91 (7.73, 20.08)
Michigan	121	124,066	29.28 (23.99, 34.56)	92	93,794	22.23 (17.37, 27.08)	41	38,522	9 (5.72, 12.29)
Minnesota	69	61,361	24.07 (17.40, 30.75)	52	48,346	19 (12.56, 25.44)	30	31,193	12.22 (6.73, 17.08)
Missouri	100	124,506	36.86 (29.81, 43.91)	78	89,163	26.62 (20.30, 32.94)	41	48,253	14.18 (9.34, 19.02)
Northern Mariana Islands	*			*			*		
Mississippi	64	57,919	37.11 (28.85, 43.36)	53	47,464	30.17 (22.23, 38.11)	25	23,983	15.1 (9.20, 20.99)
Montana	89	29,047	38.95 (30.13, 47.78)	76	20,962	28.42 (20.13, 36.71)	30	7,688	10.31 (4.77, 15.84)
North Carolina	129	184,832	30.28 (25.05, 35.51)	108	159,966	26.21 (21.09, 31.34)	53	77,116	12.55 (8.81, 16.29)
North Dakota	62	13,573	31.12 (21.86, 40.39)	48	9,519	22.26 (13.90, 30.61)	15	2,355	5.38 (2.21, 8.55)
Nebraska	60	28,286	27.16 (19.58, 34.75)	47	23,967	23.1 (15.80, 30.39)	23	11,088	10.65 (5.02, 16.28)
New Hampshire	61	25,364	30.03 (21.68, 38.37)	47	20,630	24.38 (16.34, 32.43)	21	10,687	12.56 (6.09, 19.03)
New Jersey	51	67,681	28.25 (20.05, 36.46)	42	45,062	19.21 (12.75, 25.67)	14	15,163	6.33 (2.66, 10.00)
New Mexico	86	44,221	35.82 (28.65, 42.98)	63	33,792	28.02 (21.13, 34.92)	28	15,894	12.84 (7.92, 17.76)
Nevada	62	58,445	31.8 (24.28, 39.32)	51	48,599	26.67 (19.27, 34.07)	23	21,091	11.45 (6.19, 16.72)
New York	123	145,887	27.04 (22.01, 32.08)	92	117,398	21.79 (16.92, 26.66)	36	48,799	8.9 (5.27, 12.53)
Ohio	138	158,972	27.32 (22.60, 32.04)	117	137,431	23.58 (19.04, 28.13)	41	48,938	8.39 (5.28, 11.51)
Oklahoma	76	89,254	37.03 (29.01, 45.06)	61	76,366	31.77 (23.88, 39.66)	27	39,647	16.41 (9.47, 23.34)
Oregon	86	86,486	36.97 (28.61, 45.34)	67	69,277	29.6 (21.26, 37.93)	31	31,780	13.48 (7.55, 19.41)
Pennsylvania	125	164,555	27.83 (22.81, 32.84)	100	139,312	23.73 (18.79, 28.66)	51	66,823	11.28 (7.68, 14.87)
Puerto Rico	54	15,185	25.28 (14.72, 35.84)	43	11,948	20.13 (10.17, 30.08)	*		
Rhode Island	48	10,421	22.43 (15.38, 29.48)	36	7,686	16.79 (10.48, 23.10)	14	3,477	7.42 (2.83, 12.00)
South Carolina	66	92,169	27.55 (19.57, 35.53)	59	85,112	25.65 (17.63, 33.67)	27	40,787	12.01 (5.13, 18.89)
South Dakota	64	15,031	26.88 (19.44, 34.32)	46	10,783	19.39 (12.62, 26.16)	25	7,186	12.78 (6.46, 19.10)
Tennessee	68	122,853	32.37 (24.86, 39.88)	57	113,261	30.2 (22.59, 37.81)	27	60,921	15.91 (6.21, 22.61)
Texas	308	474,006	35.79 (32.01, 39.57)	255	402,873	30.48 (26.78, 34.19)	114	197,951	14.89 (11.79, 17.99)
Utah	79	34,365	31.44 (23.85, 39.03)	63	28,104	25.71 (18.44, 32.99)	29	13,529	12.38 (7.04, 17.72)
Virginia	135	175,220	29.67 (24.30, 35.05)	104	142,320	24.18 (18.89, 29.46)	49	82,832	14.44 (9.49, 19.39)
Vermont	64	9,661	28.64 (20.20, 27.09)	48	7,587	22.36 (14.59, 30.13)	21	3,311	9.65 (4.48, 14.81)
Washington	119	168,670	37.16 (29.95, 43.13)	93	138,961	30.12 (23.02, 37.22)	44	56,595	12.17 (7.91, 16.43)
Wisconsin	82	98,925	35.42 (27.71, 43.13)	68	88,601	31.86 (24.14, 39.59)	28	45,378	16.18 (9.66, 22.71)
West Virginia	48	26,654	25.22 (17.42, 33.03)	40	21,425	20.41 (13.18, 27.64)	19	12,048	11.28 (5.29, 17.27)
Wyoming	80	11,811	31.31 (23.71, 38.91)	62	9,595	25.77 (18.50, 33.04)	28	4,306	11.36 (6.18, 16.54)

* Denotes suppressed due to unreliable estimate

#### SA

**Fig. 6:**
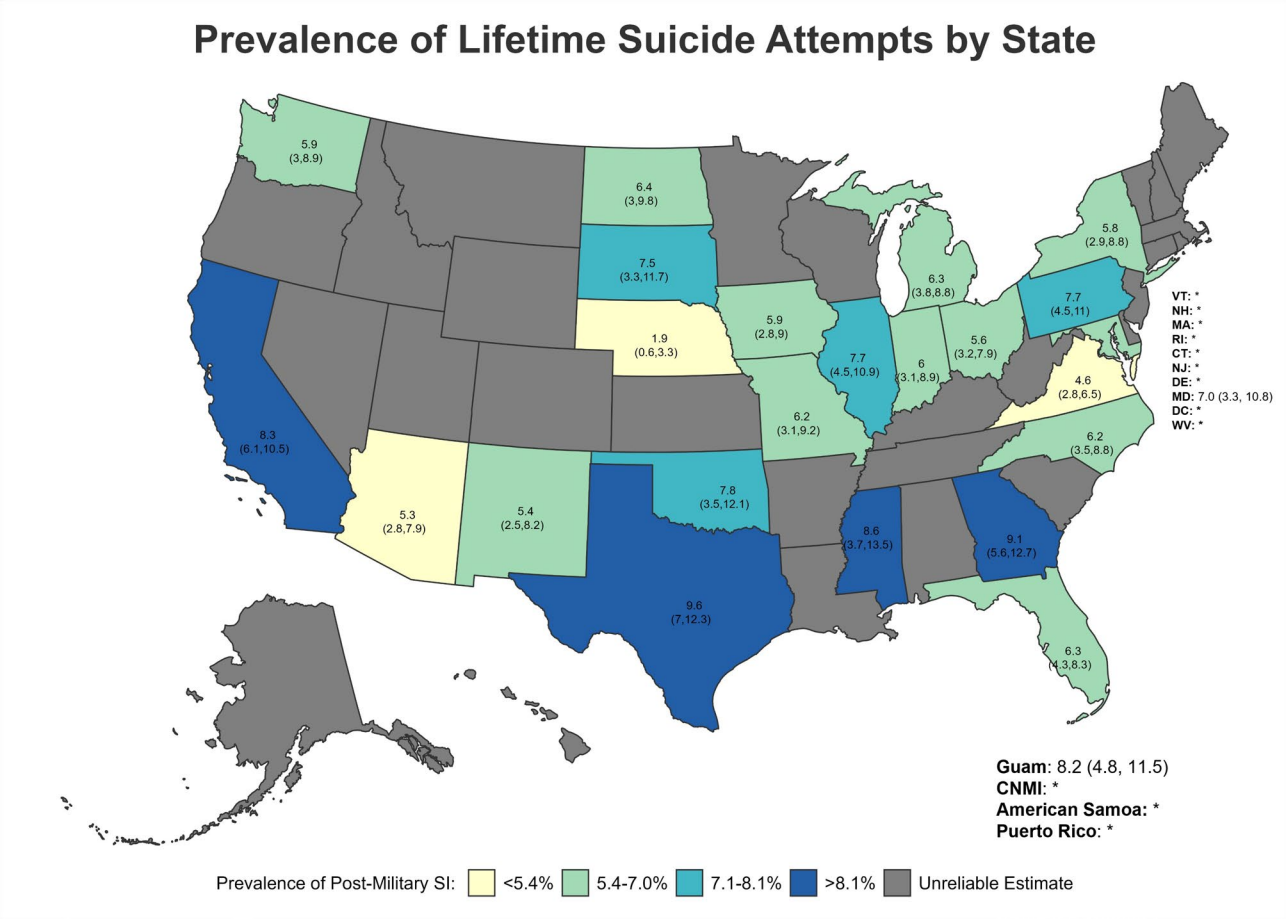
Prevalence of Lifetime Suicide Attempts by State

**Fig. 7:**
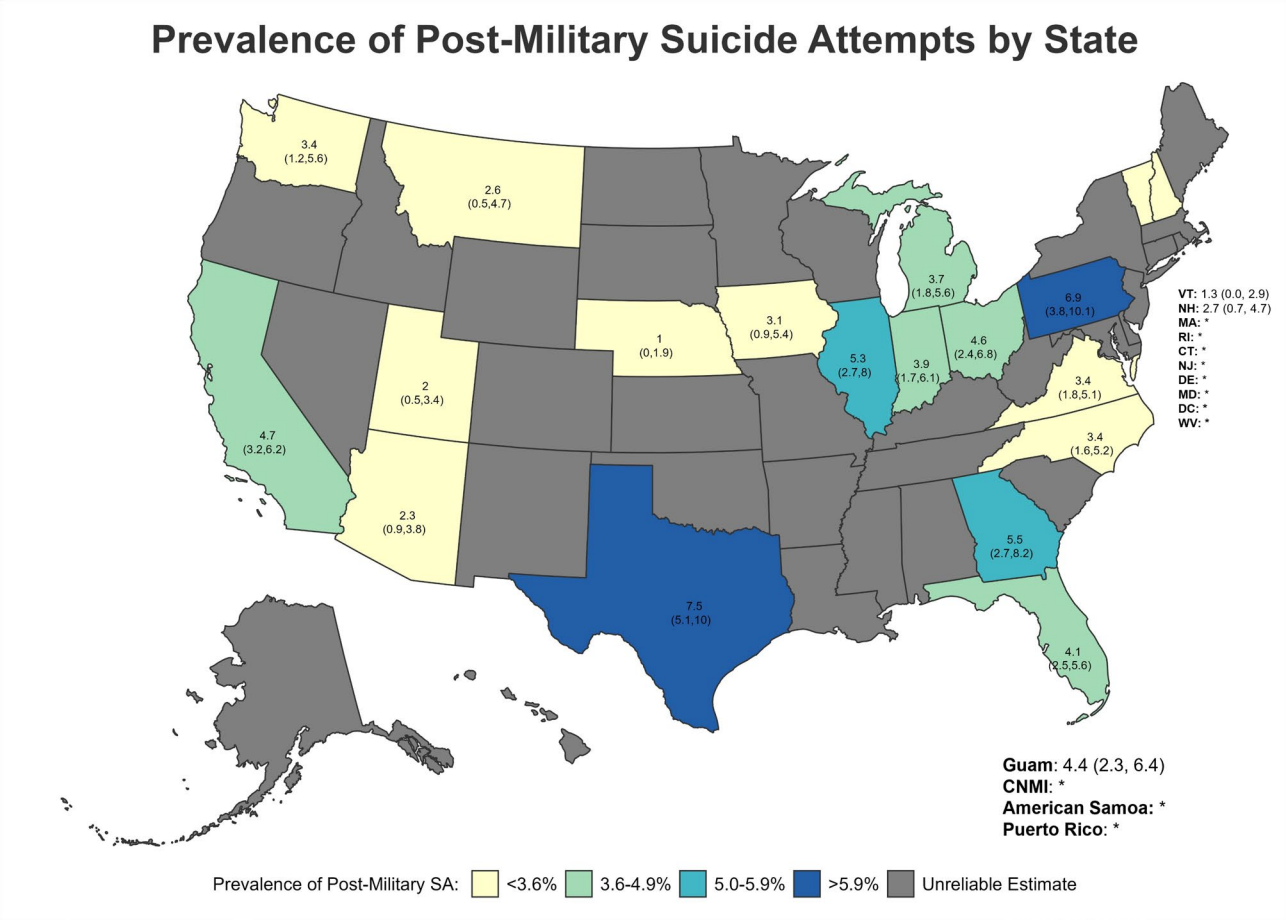
Prevalence of Post-Military Suicide Attempts by State

Suicide attempt estimates were suppressed in some states due to unreliable estimates. Among states for which estimates were considered reliable, the prevalence of lifetime SA (Fig. 6) was highest for Texas (9.63%; 95%CI = 7.01–12.25; South), Georgia (9.14%; 95%CI = 5.61–12.68; South), Mississippi (8.59%; 95%CI = 3.69–13.48; South), California (8.30%; 95%CI = 6.09–10.50; West), and Guam (8.19%; 95%CI = 4.84–11.54; PI Territories) (Table 6; Figs. 6 and 7). With regard to SA following separation from military service (Fig. 7), Texas (7.54%; 95%CI = 5.13–9.95; South), Pennsylvania (6.94%; 95%CI = 3.78–10.11; Northeast), Georgia (5.48%; 95%CI = 2.73–8.23; South), Illinois (5.32%; 95%CI = 2.70–7.95; Midwest), and California (4.69%; 95%CI = 3.19–6.18; West) had the highest reportable prevalence estimates.

**Table 6:** Prevalence estimates of lifetime and post-military suicide attempts, by state/territory

	Lifetime			Post-Military		
	Unweighted	Weighted		Unweighted	Weighted	
	N	N	% (95% CI)	N	N	% (95% CI)
Alabama	*			*		
Alaska	*			*		
American Samoa	*			*		
Arizona	23	21,524	5.35 (2.77, 7.92)	13	9,408	2.34 (0.86, 3.82)
Arkansas	*			*		
California	79	101,056	8.3 (6.09, 10.50)	48	57,597	4.69 (3.19, 6.18)
Colorado	*			*		
Connecticut	*			*		
Delaware	*			*		
District of Columbia	*			*		
Florida	65	74,162	6.33 (4.33, 8.33)	48	48,496	4.08 (2.53, 5.63)
Georgia	37	53,592	9.14 (5.61, 12.68)	23	32,362	5.48 (2.73, 8.23)
Guam	38	673	8.19 (4.84, 11.54)	23	357	4.38 (2.33, 6.43)
Hawaii	*			*		
Idaho	*			*		
Illinois	26	36,056	7.69 (4.46, 10.92)	19	24,900	5.32 (2.70, 7.95)
Indiana	22	19,757	6.03 (3.12, 8.94)	16	12,756	3.9 (1.66, 6.15)
Iowa	17	9,329	5.91 (2.83, 8.99)	9	4,920	3.13 (0.89, 5.36)
Kansas	*			*		
Kentucky	*			*		
Louisiana	*			*		
Maine	*			*		
Maryland	16	21,003	7.05 (3.27, 10.83)	*		
Massachusetts	*			*		
Michigan	35	26,457	6.28 (3.77, 8.79)	23	15,571	3.67 (1.78, 5.57)
Minnesota	*			*		
Mississippi	15	13,451	8.59 (3.69, 13.48)	*		
Missouri	21	20,812	6.16 (3.10, 9.22)	*		
Montana	*			11	1,953	2.62 (0.49, 4.74)
Nebraska	11	2,020	1.95 (0.60, 3.30)	5	1,011	0.98 (0.03, 1.93)
Nevada	*			*		
New Hampshire	*			10	2,286	2.7 (0.70, 4.70)
New Jersey	*			*		
New Mexico	16	6,627	5.37 (2.55, 8.19)	*		
New York	26	31,378	5.83 (2.85, 8.80)	*		
North Carolina	29	37,603	6.17 (3.54, 8.79)	16	20,685	3.39 (1.55, 5.23)
North Dakota	20	2,802	6.43 (3.03, 9.82)	*		
Northern Mariana Islands	*			*		
Ohio	30	32,220	5.55 (3.25, 7.86)	23	26,756	4.6 (2.41, 6.79)
Oklahoma	17	18,508	7.76 (3.47, 12.05)	*		
Oregon	*			*		
Pennsylvania	38	45,515	7.73 (4.50, 10.96)	32	41,001	6.94 (3.78, 10.11)
Puerto Rico	*			*		
Rhode Island	*			*		
South Carolina	*			*		
South Dakota	19	4,209	7.51 (3.30, 11.71)	*		
Tennessee	*			*		
Texas	77	127,586	9.63 (7.01, 12.25)	56	99,910	7.54 (5.13, 9.95)
Utah	*			9	2,126	1.96 (0.51, 3.40)
Vermont	*			3	434	1.27 (0.00, 2.85)
Virginia	27	27,390	4.64 (2.80, 6.48)	19	20,421	3.45 (1.80, 5.10)
Washington	23	26,899	5.94 (2.96, 8.93)	15	15,701	3.37 (1.16, 5.58)
West Virginia	*			*		
Wisconsin	*			*		
Wyoming	*			*		

*Suppressed due to unreliability of estimates

### Adjusted divisional comparisons

#### SI

After adjustment for covariates, the Pacific West (PR = 1.30; 95%CI = 1.09–1.53), West South Central (PR = 1.27; 95%CI = 1.08–1.49), and Mountain West (PR = 1.22; 95%CI = 1.03–1.44) divisions had significantly higher prevalence of post-military SI compared to the Mid-Atlantic (Table 7). The Pacific West (PR = 1.20; 95%CI = 1.05–1.37) and West South Central (PR = 1.18; 95%CI = 1.04–1.33) also had significantly higher prevalence of post-military SI compared to the South Atlantic. The prevalence of post-military SI in the Pacific West was also higher than in the East North Central Division (PR = 1.17; 95%CI = 1.01–1.35).

**Table 7:** Pairwise comparisons of crude and adjusted prevalence ratios for post-military suicidal ideation by census division

	Crude		Adjusted	
	PR (95% CI)	p-value	PR (95% CI)	p-value
**Division**				
**Reference: Mid-Atlantic**				
New England	1.08 (0.82, 1.34)	0.446	1.08 (0.88, 1.33)	0.455
East North Central	1.14 (0.96, 1.36)	0.141	1.11 (0.94, 1.31)	0.214
West North Central	1.11 (0.92, 1.34)	0.258	1.11 (0.92, 1.33)	0.269
South Atlantic	1.07 (0.92, 1.26)	0.380	1.08 (0.92, 1.26)	0.341
East South Central	1.19 (0.97, 1.46)	0.091	1.19 (0.98, 1.44)	0.075
West South Central	**1.31 (1.10**,** 1.56)**	**0.002**	**1.27 (1.08**,** 1.49)**	**0.004**
Mountain West	**1.27 (1.06**,** 1.51)**	**0.008**	**1.22 (1.03**,** 1.44)**	**0.024**
Pacific West	**1.31 (1.10**,** 1.56)**	**0.002**	**1.30 (1.09**,** 1.53)**	**0.003**
**Reference: New England**				
East North Central	1.05 (0.87, 1.27)	0.601	1.03 (0.85, 1.24)	0.780
West North Central	1.03 (0.84, 1.25)	0.787	1.02 (0.84, 1.25)	0.814
East South Central	1.10 (0.89, 1.36)	0.384	1.10 (0.89, 1.36)	0.376
West South Central	**1.21 (1.01**,** 1.45)**	**0.043**	1.17 (0.97, 1.41)	0.092
Mountain West	1.17 (0.97, 1.41)	0.101	1.13 (0.93, 1.36)	0.228
Pacific West	**1.21 (1.00**,** 1.46)**	**0.046**	1.20 (0.99, 1.45)	0.063
**Reference: East North Central**				
East South Central	1.05 (0.87, 1.26)	0.621	1.07 (0.90, 1.27)	0.433
West South Central	1.15 (1.00, 1.33)	0.056	1.14 (0.99, 1.31)	0.062
Mountain West	1.11 (0.96, 1.29)	0.157	1.10 (0.95, 1.27)	0.225
Pacific West	1.15 (0.99, 1.34)	0.062	**1.17 (1.01**,** 1.35)**	**0.040**
**Reference: West North Central**				
East North Central	1.02 (0.87, 1.20)	0.790	1.00 (0.85, 1.18)	0.975
East South Central	1.07 (0.88, 1.30)	0.495	1.07 (0.89, 1.29)	0.456
West South Central	1.18 (1.00, 1.38)	0.049	1.14 (0.98, 1.34)	0.095
Mountain West	1.14 (0.97, 1.34)	0.124	1.10 (0.93, 1.30)	0.267
Pacific West	1.18 (1.00, 1.39)	0.052	1.17 (0.99, 1.38)	0.064
**Reference: South Atlantic**				
New England	1.01 (0.85, 1.20)	0.921	1.00 (0.84, 1.20)	0.965
East North Central	1.06 (0.93, 1.21)	0.383	1.03 (0.91, 1.17)	0.640
West North Central	1.04 (0.89, 1.20)	0.634	1.03 (0.89, 1.19)	0.713
East South Central	1.11 (0.94, 1.32)	0.228	1.10 (0.94, 1.29)	0.224
West South Central	**1.22 (1.07**,** 1.39)**	**0.002**	**1.18 (1.04**,** 1.33)**	**0.01**
Mountain West	**1.18 (1.03**,** 1.35)**	**0.015**	1.13 (0.99, 1.29)	0.076
Pacific West	**1.22 (1.07**,** 1.40)**	**0.003**	**1.20 (1.05**,** 1.37)**	**0.006**
**Reference: East South Central**				
West South Central	1.10 (0.92, 1.32)	0.302	1.07 (0.90, 1.26)	0.452
Mountain West	1.06 (0.89, 1.28)	0.511	1.02 (0.86, 1.22)	0.796
Pacific West	1.10 (0.92, 1.32)	0.307	1.09 (0.92, 1.30)	0.335
**Reference: West South Central**				
Pacific West	1.00 (0.87, 1.16)	0.989	1.02 (0.89, 1.18)	0.767
**Reference: Mountain West**				
West South Central	1.03 (0.89, 1.20)	0.656	1.04 (0.90, 1.20)	0.577
Pacific West	1.03 (0.89, 1.20)	0.656	1.06 (0.92, 1.24)	0.416

Note: While all pairwise comparisons were calculated, results are only presented for crude prevalence ratios where the reference group has the lower prevalence for ease of interpretation. Bold text indicates significant findings at the *p* <.05 level

Regarding past-year SI (Table 8), after adjustment, the East South Central (PR = 1.49; 95%CI = 1.08–2.06), West North Central (PR = 1.43; 95%CI = 1.06–1.93), West South Central (PR = 1.40; 95%CI = 1.05–1.86), and South Atlantic (PR = 1.36; 95%CI = 1.05–1.77) had significantly higher prevalence compared to the Mid-Atlantic.

**Table 8:** Pairwise comparisons of crude and adjusted prevalence ratios for past-year suicidal ideation by census division

	Crude		Adjusted	
	PR (95% CI)	p-value	PR (95% CI)	p-value
**Division**				
**Reference: Mid-Atlantic**	Ref		Ref	
New England	1.07 (0.76, 1.52)	0.685	1.09 (0.77, 1.55)	0.613
East North Central	1.15 (0.86, 1.54)	0.344	1.14 (0.86, 1.52)	0.364
West North Central	**1.39 (1.02**,** 1.88)**	**0.035**	**1.43 (1.06**,** 1.93)**	**0.021**
South Atlantic	**1.41 (1.08**,** 1.84)**	**0.011**	**1.36 (1.05**,** 1.77)**	**0.019**
East South Central	**1.47 (1.04**,** 2.06)**	**0.027**	**1.49 (1.08**,** 2.06)**	**0.016**
West South Central	**1.48 (1.11**,** 1.97)**	**0.008**	**1.40 (1.05**,** 1.86)**	**0.021**
Mountain West	**1.43 (1.06**,** 1.93)**	**0.02**	1.34 (1.00, 1.81)	0.052
Pacific West	**1.43 (1.07**,** 1.92)**	**0.016**	1.31 (0.98, 1.76)	0.07
**Reference: New England**				
East North Central	1.07 (0.78, 1.47)	0.664	1.04 (0.76, 1.43)	0.789
West North Central	1.29 (0.93, 1.78)	0.122	1.30 (0.94, 1.81)	0.113
South Atlantic	1.31 (0.98, 1.75)	0.065	1.25 (0.93, 1.67)	0.137
East South Central	1.36 (0.95, 1.95)	0.089	1.36 (0.96, 1.92)	0.083
West South Central	**1.37 (1.01**,** 1.87)**	**0.045**	1.28 (0.94, 1.75)	0.124
Mountain West	1.33 (0.96, 1.83)	0.083	1.23 (0.89, 1.69)	0.216
Pacific West	1.33 (0.98, 1.82)	0.071	1.20 (0.87, 1.65)	0.267
**Reference: East North Central**				
West North Central	1.20 (0.93, 1.57)	0.166	1.25 (0.96, 1.62)	0.094
South Atlantic	1.22 (0.99, 1.52)	0.068	1.19 (0.97, 1.48)	0.100
East South Central	1.27 (0.94, 1.73)	0.119	1.30 (0.98, 1.73)	0.070
West South Central	**1.28 (1.00**,** 1.64)**	**0.048**	1.22 (0.96, 1.56)	0.099
Mountain West	1.24 (0.96, 1.60)	0.104	1.17 (0.91, 1.51)	0.215
Pacific West	1.24 (0.97, 1.59)	0.085	1.15 (0.89, 1.48)	0.280
**Reference: West North Central**				
South Atlantic	1.02 (0.81, 1.28)	0.895	0.96 (0.76, 1.21)	0.709
East South Central	1.06 (0.77, 1.45)	0.727	1.04 (0.78, 1.41)	0.779
West South Central	1.06 (0.82, 1.38)	0.634	0.98 (0.76, 1.27)	0.886
Mountain West	1.03 (0.79, 1.35)	0.838	0.94 (0.72, 1.23)	0.658
Pacific West	1.03 (0.80, 1.34)	0.810	0.92 (0.70, 1.20)	0.541
**Reference: South Atlantic**				
East South Central	1.04 (0.79, 1.37)	0.774	1.09 (0.84, 1.41)	0.508
West South Central	1.05 (0.85, 1.30)	0.661	1.03 (0.83, 1.26)	0.811
Mountain West	1.01 (0.81, 1.27)	0.911	0.98 (0.79, 1.23)	0.883
Pacific West	1.02 (0.82, 1.26)	0.879	0.96 (0.77, 1.20)	0.725
**Reference: East South Central**				
West South Central	1.01 (0.75, 1.36)	0.965	0.94 (0.71, 1.25)	0.669
**Reference: Mountain West**				
East South Central	1.03 (0.75, 1.40)	0.861	1.11 (0.83, 1.49)	0.491
West South Central	1.04 (0.80, 1.34)	0.79	1.04 (0.81, 1.34)	0.743
**Reference: Pacific West**				
East South Central	1.02 (0.76, 1.39)	0.876	1.13 (0.85, 1.52)	0.396
West South Central	1.03 (0.81, 1.32)	0.804	1.07 (0.83, 1.37)	0.61
Mountain West	1.00 (0.77, 1.29)	0.977	1.02 (0.79, 1.33)	0.865

Note: While all pairwise comparisons were calculated, results are only presented for crude prevalence ratios where the reference group has the lower prevalence for ease of interpretation. Bold text indicates significant findings at the *p* <.05 level

#### SA

After adjustment for covariates, the West South Central division had a higher prevalence of post-military SA compared to the Mountain West (PR = 1.85; 95%CI = 1.21–2.83), West North Central (PR = 1.84; 95%CI = 1.19–2.83), New England (PR = 1.77; 95%CI = 1.10–2.85), Pacific West (PR = 1.47; 95%CI = 1.00–2.16), and South Atlantic (PR = 1.46; 95%CI = 1.04–2.04) (Table 9).

**Table 9:** Pairwise comparisons of crude and adjusted prevalence ratios for post-military suicide attempts by census division

	Crude		Adjusted	
	PR (95% CI)	p-value	PR (95% CI)	p-value
**Division**				
**Reference: Mid-Atlantic**	Ref		Ref	
East South Central	1.15 (0.69, 1.91)	0.597	1.04 (0.63, 1.73)	0.868
West South Central	1.30 (0.85, 1.99)	0.233	1.19 (0.78, 1.83)	0.422
**Reference: New England**				
Mid-Atlantic	1.49 (0.90, 2.47)	0.122	1.48 (0.87, 2.53)	0.149
East North Central	1.38 (0.88, 2.16)	0.167	1.31 (0.82, 2.09)	0.263
West North Central	1.01 (0.61, 1.67)	0.964	0.96 (0.56, 1.64)	0.887
South Atlantic	1.28 (0.83, 1.98)	0.272	1.21 (0.76, 1.92)	0.419
East South Central	1.71 (1.00, 2.93)	0.051	1.55 (0.90, 2.66)	0.116
West South Central	**1.93 (1.22**,** 3.06)**	**0.005**	**1.77 (1.10**,** 2.85)**	**0.02**
Pacific West	1.27 (0.80, 2.01)	0.311	1.20 (0.73, 1.97)	0.465
**Reference: East North Central**				
Mid-Atlantic	1.08 (0.71, 1.64)	0.711	1.13 (0.74, 1.74)	0.569
East South Central	1.24 (0.79, 1.96)	0.354	1.18 (0.76, 1.85)	0.462
West South Central	1.40 (0.98, 2.02)	0.068	1.35 (0.94, 1.93)	0.1
**Reference: West North Central**				
Mid-Atlantic	1.47 (0.92, 2.36)	0.109	1.54 (0.94, 2.53)	0.088
East North Central	1.36 (0.90, 2.06)	0.148	1.36 (0.88, 2.09)	0.163
South Atlantic	1.26 (0.85, 1.88)	0.253	1.26 (0.83, 1.92)	0.284
East South Central	**1.69 (1.02**,** 2.81)**	**0.043**	1.61 (0.96, 2.68)	0.069
West South Central	**1.91 (1.25**,** 2.92)**	**0.003**	**1.84 (1.19**,** 2.83)**	**0.006**
Pacific West	1.25 (0.82, 1.91)	0.297	1.25 (0.79, 1.97)	0.337
**Reference: South Atlantic**				
Mid-Atlantic	1.17 (0.78, 1.74)	0.455	1.22 (0.81, 1.85)	0.338
East North Central	1.08 (0.77, 1.50)	0.663	1.08 (0.78, 1.50)	0.646
East South Central	1.34 (0.86, 2.08)	0.198	1.28 (0.84, 1.94)	0.25
West South Central	**1.51 (1.07**,** 2.13)**	**0.019**	**1.46 (1.04**,** 2.04)**	**0.028**
**Reference: East South Central**				
West South Central	1.13 (0.71, 1.80)	0.608	1.14 (0.73, 1.78)	0.558
**Reference: Mountain West**				
Mid-Atlantic	1.49 (0.93, 2.39)	0.102	1.55 (0.95, 2.53)	0.078
New England	1.00 (0.60, 1.65)	0.993	1.05 (0.62, 1.77)	0.862
East North Central	1.37 (0.90, 2.08)	0.137	1.37 (0.90, 2.09)	0.141
West North Central	1.01 (0.63, 1.62)	0.968	1.01 (0.62, 1.64)	0.974
South Atlantic	1.27 (0.85, 1.90)	0.236	1.27 (0.84, 1.91)	0.255
East South Central	**1.70 (1.02**,** 2.84)**	**0.04**	1.62 (0.98, 2.68)	0.06
West South Central	**1.93 (1.26**,** 2.95)**	**0.003**	**1.85 (1.21**,** 2.83)**	**0.005**
Pacific West	1.26 (0.83, 1.93)	0.279	1.26 (0.81, 1.96)	0.304
**Reference: Pacific West**				
Mid-Atlantic	1.17 (0.77, 1.80)	0.458	1.23 (0.79, 1.93)	0.365
East North Central	1.09 (0.76, 1.56)	0.657	1.09 (0.74, 1.59)	0.666
South Atlantic	1.01 (0.72, 1.42)	0.964	1.01 (0.70, 1.46)	0.973
East South Central	1.35 (0.85, 2.15)	0.208	1.29 (0.80, 2.06)	0.294
West South Central	**1.52 (1.05**,** 2.21)**	**0.026**	**1.47 (1.00**,** 2.16)**	**0.0499**

Note: While all pairwise comparisons were calculated, results are only presented for crude prevalence ratios where the reference group has the lower prevalence for ease of interpretation. Bold text indicates significant findings at the *p* <.05 level

## Discussion

Our findings provide the first in-depth overview of geographic differences in prevalence of SI and SA among U.S. Veterans. The variability observed at state, divisional and regional levels supports the need for nuanced, detailed surveillance efforts. Further, consistent with the VA’s public health approach to suicide prevention, these findings support the importance of targeted efforts within areas at greatest risk [20].

Indeed, state and divisional findings underscore the importance of granular geographic estimates. For example, the West had the highest regional prevalence of post-military SI (28.7%), and the Pacific West maintained the highest prevalence of post-military SI at the division level (29.1%); however, the West South Central division (within the South region) had a similar prevalence of post-military SI (29.1%). For post-military SA, while PI Territories and the South had the highest regional prevalence (5.7% and 5.5%, respectively), risk in the South was more concentrated in the West South Central (6.9%) and East South Central (6.1%) divisions relative to the South Atlantic (4.6%). Finally, in comparing SI and SA findings by region and division, the Northeast region had the lowest prevalence of SI across timeframes as well as lifetime SA, yet SI prevalence estimates were higher in New England than the Mid-Atlantic for all timepoints and SA estimates were lower in New England than the Mid-Atlantic for all timeframes. State-level estimates provided a further nuanced understanding of geographical differences. All five states with the highest lifetime SI prevalence, and two of the five states with the highest post-military SI prevalence, were in the West, whereas three of the five states with the highest past-year SI prevalence were in the South. Elucidating such differences is critical given the import of targeting prevention efforts to more recent suicidal behavior.

Considering our findings in the context of geographic differences in suicide rates and emergency department visits for SI and SA highlights both consistencies and discrepancies. Specifically, in 2022, among Veterans, the West had the highest regional suicide rate (40.40/100,000), followed by the Midwest (35.70/100,000), South (34.30/100,000), and Northeast (25.00/100,000); a pattern mirrored in the general population [4]. This is consistent with our findings that, of those four regions, the West had the highest prevalence of lifetime and post-military SI, followed by the Midwest and South, with the Northeast having the lowest prevalence for all SI outcomes. Additionally, in the general population, the West had the highest prevalence of emergency department visits for SI or SA [21]. However, the West had the lowest prevalence of post-military SA in our analysis. This discrepancy may be due to a number of factors, including the high prevalence of firearm suicides in the West (71.4% of Veteran suicides in 2022), mental health stigma, and access to healthcare [22–27]. Indeed, the five states with the highest Veteran suicide rates in 2022—Montana, Utah, Nevada, Oregon, and Idaho—have high rates of both firearm ownership and firearm suicides [4, 22]. Moreover, there are highly rural areas within these states. Thus, relatively lower prevalence of suicide attempts in these states, in the context of high suicide rates, may be due to the use of highly lethal suicide methods, physical or cultural barriers to accessing mental health care, or deterrents to reporting suicidal ideation and behavior (e.g., stigma).

Relatedly, although we did not examine factors driving geographic differences in SI and SA, healthcare access, rurality, and mental health stigma may contribute to these differences [23–27] and will be important to examine in future research. These factors also may intersect to impact suicide risk. For example, healthcare access is more limited in rural areas (i.e., 62% and 78% of rural and highly rural Veterans, respectively, live in areas with greater than one hour travel time to the nearest VA health care facility care) and the likelihood of living in a rural area varies by region; in our analysis, double the proportion of Veterans in the Midwest lived in a rural area compared to the West [28]. Further, it is estimated that between 1 and 7 million Veterans do not have reliable internet access to receive care via telehealth, many of whom are located in rural areas [29]. Notably, lack of access to healthcare is directly associated with suicidal ideation in Veterans [30]. The impact of healthcare use is further compounded by stigma, which can hinder help-seeking [27, 31]. Areas with a strong “culture of honor” (i.e., cultures which emphasize defense of the reputation of oneself and family), such as the South (e.g., Texas) and West may also impact help-seeking behaviors due to reputation concerns, which may contribute to higher levels of suicidal ideation among Veterans in these areas, particularly among men [32].

Additionally, Veterans in rural areas have higher firearm ownership and differing attitudes towards firearms [23, 24]. Moreover, states in the Mountain West and West South Central, including Texas, as well as Maine (New England), have less stringent firearm laws and no waiting period for handgun purchase [33]. Additionally, states in the Mountain West, such as Colorado, Wyoming, and Montana, and the West South Central, such as Texas, have the highest proportions of Veterans living in rural and highly rural areas [28, 29]. Indeed, Veterans in rural areas have higher suicide rates and are more likely to use firearms (which have a highest case fatality rate) as their suicide method [23, 25, 34]. These factors may help to explain why specific regions that are highly rural (e.g., Mountain West) had a high prevalence of SI, but lower prevalence of non-fatal SA relative to other divisions, as the majority of SA using firearms result in death [34]. Additionally, travel time to a hospital is associated with increased fatality from suicide attempts of any methods, and longer transport times after any type of firearm injury is associated with increased mortality from the injuries [35, 36]. Shorter travel times to Level I or II trauma centers in the Northeast compared to other regions may thus partially explain why Veterans in the Northeast had the highest proportion of firearm use in non-fatal suicide attempts compared to other regions [37]; Veterans in the Northeast who attempt suicide using a firearm may be more likely to have access to life-saving emergency care. While future research to elucidate the combination of factors driving geographical variations in NF-SSDV is critical, targeted, community-level, upstream suicide prevention efforts are needed.

Socioeconomic stressors are also important to consider when examining regional differences in suicide risk. Homelessness, financial problems (including debt), food insecurity, and job loss are all associated with increased risk for suicide, suicidal ideation, and suicide attempts in Veterans [30, 38–40]. States in the South, such as Texas and Oklahoma, and West, such as New Mexico, California, and Oregon, have high poverty rates and high food insecurity compared to national levels, as well as high rates of Veteran unemployment [41, 42]. Furthermore, Pacific West states (e.g., California, Oregon, and Washington) and Texas have a high prevalence of unhoused Veterans [43]. Food insecurity and unemployment are also more common in rural areas [44, 45]. These socioeconomic factors may contribute to high prevalence of SI and SA in these areas.

This study has limitations to consider. First, SI and SA data were collected by self-report, which may be subject to recall and presentation bias, leading to underestimates [46, 47]. Additionally, though ASCEND aimed to collect data from a representative sample of all US Veterans, some state/territory sample sizes were small, particularly for estimating SA prevalence, leading to suppression of unreliable estimates. Recurring survey administration will facilitate combining data over time to produce more reliable and precise state-level estimates [12]. The response rate in both the main sample (19.2%) and the PI sample (21.6%) is comparable to other national studies of Veterans, including the Million Veteran Program (13%) and the Veterans Metrics Initiative (23%) [48, 49]. Nevertheless, there is potential for non-response bias. We have attempted to minimize such potential through purposeful sampling and mixed-mode data collection. Furthermore, our population sampling frame allowed for assessment of non-response and the development of non-response weights to correct for potential non-response effects. However, some non-response bias is still possible. Moreover, unstably housed Veterans are often harder to recruit for survey studies, which require a stable mailing address and social drivers of health related to transitional living arrangements may impact findings. Veterans residing in the U.S. Virgin Islands were not included and will be included in future waves of ASCEND. Finally, there is a potential for differential survivor bias to impact regional comparisons given that Veterans must be alive to be enrolled in the ASCEND study and suicide rates vary geographically (e.g., highest in the West). This underscores the need for primary prevention efforts for suicide in the Veteran population, as Veterans—both in general and in the West specifically—are more likely than the general population to use highly lethal means for suicide (i.e., firearms), increasing the likelihood of dying on their first suicide attempt [50].

Despite these limitations, these novel findings, situated in the context of extant literature, indicate that suicide prevention strategies for Veterans cannot use a one-size-fits-all approach. Developing and implementing tailored prevention strategies that consider geographic differences in suicide risk is requisite. For example, knowledge of geographic differences in suicide methods used in suicidal self-directed violence can be applied to inform region-specific lethal means safety efforts. Future research that illuminates regional differences in drivers of SI and SA will also be essential to targeted prevention efforts in each region. Finally, continued efforts to partner with communities in different regions are likely to be integral to implementing suicide prevention strategies that are responsive to the needs and unique considerations in each region [20, 51, 52].

## Electronic supplementary material

Supplementary Material 1

Supplementary Material 2

Supplementary Material 3

## Data Availability

Data cannot be shared publicly because of privacy and confidentiality requirements for this study. Specifically, institutional restrictions prohibit us from sharing this data publicly, as data from this study include potentially sensitive information from U.S. military Veterans. Thus, we do not have approval by our regulatory authority (the VA Rocky Mountain Regional VA Research and Development Committee) to share de-identified data publicly for this study. Rather, de-identified data can be accessed with a Data Use Agreement and verification of IRB approval from the requestor. Data are available from the VA R&D Committee (contact via 303-399-8020) for researchers who meet the criteria for access to confidential data. Our local regulatory authority (the VA Rocky Mountain Regional VA Research & Development Committee) is available to review any such requests.

## Footnotes

1 The denominator for Veteran suicide rates is the estimated number of living Veterans as of July 1 of the year of analysis as estimated by the Veteran Population Projection Model 2020 (VetPop2020) [1].

2 Estimates were computed independently for the main and PI Territories samples as differences in the sample design preclude and weighting preclude combining across these samples.

3 Northeast: Connecticut, Maine, Massachusetts, New Hampshire, New Jersey, New York, Pennsylvania, Rhode Island, Vermont; Midwest: Illinois, Indiana, Iowa, Kansas, Michigan, Minnesota, Missouri, Nebraska, North Dakota, Ohio, South Dakota, Wisconsin; South: Alabama, Arkansas, Delaware, District of Colombia, Florida, Georgia, Louisiana, Kentucky, Maryland, Mississippi, North Carolina, Oklahoma, South Carolina, Tennessee, Virginia, West Virginia; West: Alaska, Arizona, California, Colorado, Hawaii, Idaho, Montana, Nevada, New Mexico, Oregon, Utah, Washington, Wyoming.

4 New England: Connecticut, Maine, Massachusetts, New Hampshire, Rhode Island, Vermont; Mid-Atlantic: New Jersey, New York, Pennsylvania; East North Central: Illinois, Indiana, Michigan, Ohio, Wisconsin; West North Central: Iowa, Kansas, Minnesota, Missouri, Nebraska, North Dakota, South Dakota; South Atlantic: Delaware, District of Colombia, Florida, Georgia, Maryland, North Carolina, South Carolina, Virginia, West Virginia; East South Central: Alabama, Kentucky, Mississippi, Tennessee; West South Central: Arkansas, Louisiana, Oklahoma, Texas; Mountain West: Arizona, Colorado, Idaho, Montana, Nevada, New Mexico, Utah, Wyoming; Pacific West: Alaska, California, Hawaii, Oregon, Washington.

5 RUCA Codes 1.0, 1.1, 2.0, 2.1, 3.0, 4.1, 5.1, 7.1, 8.1, and 10.1 were classified as Urban. All others were classified as rural. The Pacific Island Territories do not have assigned RUCA codes and are thus not assigned rurality.

## References

[1] Department of Veterans Affairs. 2024 National Veteran Suicide Prevention Annual Report. [Internet] 2024. Available from: https://www.mentalhealth.va.gov/docs/data-sheets/2024/2024_Annual_Report_Methods_Summary_508.pdf

[2] Department of Veterans Affairs. 2024 National Veteran Suicide Prevention Annual Report. [Internet] 2024. Available from: https://www.mentalhealth.va.gov/docs/data-sheets/2024/2024-Annual-Report-Part-2-of-2_508.pdf

[3] United States Census Bureau. Sex by Age by Veteran Status for the Civilian Population 18 Years and Over. [Internet]. 2024. Available from: https://data.census.gov/table/ACSDT1Y2022.B21001?q=Veterans&y=2022

[4] Department of Veterans Affairs. 2001–2021 State Sheets Suicide Data Appendix. [Internet]. 2024. Available from: https://www.mentalhealth.va.gov/docs/data-sheets/2022/VA_State_Sheets_2001-2022_Appendix_508.xlsx

[5] Steelesmith DL, Fontanella CA, Campo JV, Bridge JA, Warren KL, Root ED. Contextual factors associated with County-Level suicide rates in the united States, 1999 to 2016. JAMA Netw Open. 2019;2(9):e1910936. 10.1001/jamanetworkopen.2019.10936. 31490540 10.1001/jamanetworkopen.2019.10936 PMC6735416

[6] Rogerson P, Yang J, Bagchi-Sen S. Recent geographic patterns in suicide in the united States. GeoJournal. 2024;89(1):19.

[7] Nock MK, Borges G, Bromet EJ, Cha CB, Kessler RC, Lee S. Suicide and suicidal behavior. Epidemiol Rev. 2008;30(1):133. 18653727 10.1093/epirev/mxn002 PMC2576496

[8] Hein TC, Cooper SA, McCarthy JF. Mortality following non-fatal suicide attempts by veterans in veterans health administration care. Suicide Life-Threatening Behav. 2021;52(2):222–30. 10.1111/sltb.12811. 10.1111/sltb.12811 34816474

[9] Brown BA, Goodman FR, Pietrzak RH, Rottenberg J. Psychological well-being in US veterans with non-fatal suicide attempts: A multi-cohort population-based study. J Affect Disord. 2022;314:34–43. 35803391 10.1016/j.jad.2022.07.003

[10] Peterson C, Haileyesus T, Stone DM. Economic cost of US suicide and non-fatal self-harm. Am J Prev Med. 2024;67(1):129–33. 38479565 10.1016/j.amepre.2024.03.002 PMC11193601

[11] Peterson A, Bozzay M, Bender A, Monahan M, Chen J. Those left behind: A scoping review of the effects of suicide exposure on veterans, service members, and military families. Death Stud. 2022;46(5):1176–85. 32762420 10.1080/07481187.2020.1802628 PMC8162890

[12] Hoffmire CA, Barnes SM, Holliday R, Kittel JA, Schneider AL, Brenner LA, Tock JL, Monteith LL. Non-fatal suicidal self-directed violence among U.S. Veterans (2022): The Assessing Social and Community Environments with National Data (ASCEND) for Veteran Suicide Prevention Study. Am J Epidemiol. 2024 Dec 17:kwae461. 10.1093/aje/kwae461. Epub ahead of print. PMID: 39694860. 10.1093/aje/kwae461 PMC13343375 39694860

[13] Monteith LL, Kittel JA, Holliday R, Schneider AL, Herring-Nathan E, Krishnamurti LS, Brenner LA, Hoffmire CA. Suicidal ideation and non-fatal suicidal self-directed violence prevalence and associations among veterans residing in U.S. Pacific Island territories: Guam, American Samoa, and the Northern Mariana Islands. (manuscript under review). 10.1371/journal.pone.0326533 PMC12176182 40531952

[14] Hauser G, United States Veterans Eligibility Trends and Statistics (USVETS). : A New Data Source with Socioeconomic Variables. VA HSR&D VIReC Database and Methods Seminar. May 6, 2019. Available at: https://www.hsrd.research.va.gov/for_researchers/cyber_seminars/archives/video_archive.cfm?SessionID=3626

[15] Posner K, Brown GK, Stanley B, Brent DA, Yershova KV, Oquendo MA, Currier GW, Melvin GA, Greenhill L, Shen S, Mann JJ. The Columbia-Suicide severity rating scale: initial validity and internal consistency findings from three multisite studies with adolescents and adults. Am J Psychiatry. 2011;168(12):1266–77. 10.1176/appi.ajp.2011.10111704. 22193671 10.1176/appi.ajp.2011.10111704 PMC3893686

[16] Hoffmire CA, Mohatt NV, Holliday R, Barnes SM, Brenner LA, Monteith LL. ASCEND for veteran suicide prevention: enhancing surveillance to save lives. Psychiatry Res. 2022;310:114432. 35150969 10.1016/j.psychres.2022.114432

[17] U.S. Census Bureau. Census Regions and Divisions [Internet]. 1984 Available from: https://www2.census.gov/geo/pdfs/maps-data/maps/reference/us_regdiv.pdf

[18] Parker JD, Talih M, Malec DJ, Beresovsky V, Carroll M, Gonzalez JF, Hamilton BE, Ingram DD, Kochanek K, McCarty F, Moriarity C, Shimizu I, Strashny A, Ward BW. National center for health statistics data presentation standards for proportions. Vital Health Stat 2. 2017;(175):1–22. PMID: 30248016. 30248016

[19] Talbot D, Mésidor M, Chiu Y, Simard M, Sirois C. An alternative perspective on the robust Poisson method for estimating risk or prevalence ratios. Epidemiology. 2023;34(1):1–7. 10.1097/EDE.0000000000001544. 36125349 10.1097/EDE.0000000000001544

[20] 2018; Department of Veterans Affairs. National Strategy for Preventing Veteran Suicide, Washington DC. Department of Veteran Affairs. [Internet]. 2018. Available from: https://www.mentalhealth.va.gov/suicide_prevention/docs/Office-of-Mental-Health-and-Suicide-Prevention-National-Strategy-for-Preventing-Veterans-Suicide.pdf

[21] Zwald ML, Holland KM, Annor FB, Kite-Powell A, Sumner SA, Bowen DA, Vivolo-Kantor AM, Stone DM, Crosby AE. Syndromic surveillance of suicidal ideation and self-directed violence—United States, January 2017–December 2018. MMWR Morb Mortal Wkly Rep. 2020;69(4):103–8. 31999688 10.15585/mmwr.mm6904a3 PMC7004405

[22] RAND. Gun Ownership in America. [Internet]. Available from: https://www.rand.org/research/gun-policy/gun-ownership.html

[23] McCarthy JF, Blow FC, Ignacio RV, Ilgen MA, Austin KL, Valenstein M. Suicide among patients in the veterans affairs health system: rural-urban differences in rates, risks, and methods. Am J Public Health. 2012;102(Suppl 1):S111–7. 10.2105/AJPH.2011.300463. 22390583 10.2105/AJPH.2011.300463 PMC3496440

[24] Monteith LL, Wendleton L, Bahraini NH, Matarazzo BB, Brimner G, Mohatt NV. Together with veterans: VA National strategy alignment and lessons learned from Community-Based suicide prevention for rural veterans. Suicide Life Threat Behav. 2020;50(3):588–600. 10.1111/sltb.12613. 31950557 10.1111/sltb.12613

[25] Denneson LM, Bollinger MJ, Phillips R, Chen JI, Carlson KF. County characteristics and veteran suicide in the united States, 2011–2018. Am J Prev Med. 2024;67(5):689–97. 10.1016/j.amepre.2024.06.011. 38906428 10.1016/j.amepre.2024.06.011

[26] Gujral K, Van Campen J, Jacobs J, Kimerling R, Blonigen D, Zulman DM. Mental health service use, suicide behavior, and emergency department visits among rural US veterans who received Video-Enabled tablets during the COVID-19 pandemic. JAMA Netw Open. 2022;5(4):e226250. 10.1001/jamanetworkopen.2022.6250. 35385088 10.1001/jamanetworkopen.2022.6250 PMC8987904

[27] Monteith LL, Smith NB, Holliday R, Dorsey Holliman BA, LoFaro CT, Mohatt NV. We’re afraid to say suicide: stigma as a barrier to implementing a Community-Based suicide prevention program for rural veterans. J Nerv Ment Dis. 2020;208(5):371–6. 10.1097/NMD.0000000000001139. 31895224 10.1097/NMD.0000000000001139

[28] West AN, Lee RE, Shambaugh-Miller MD, Bair BD, Mueller KJ, Lilly RS, Kaboli PJ, Hawthorne K. Defining rural for veterans’ health care planning. J Rural Health 2010 Fall;26(4):301–9. 10.1111/j.1748-0361.2010.00298.x 10.1111/j.1748-0361.2010.00298.x 21029164

[29] Department of Veterans Affairs, Office of Rural Health. 2023 THRIVE Annual Report. [Internet]. 2024. Available at: https://www.ruralhealth.va.gov/docs/ORH-THRIVE_Annual-Report-Fiscal-Year-2023_508c.pdf

[30] Blosnich JR, Montgomery AE, Dichter ME, Gordon AJ, Kavalieratos D, Taylor L, Ketterer B, Bossarte RM. Social determinants and military Veterans’ suicide ideation and attempt: a cross-sectional analysis of electronic health record data. J Gen Intern Med. 2020;35:1759–67. 31745856 10.1007/s11606-019-05447-z PMC7280399

[31] Schroeder S, Tan CM, Urlacher B, Heitkamp T. The role of rural and urban geography and gender in community stigma around mental illness. Health Educ Behav. 2021;48(1):63–73. 10.1177/1090198120974963. 33218261 10.1177/1090198120974963

[32] Foster S, Carvallo M, Lee J, Bernier I. Honor and seeking mental health services: the roles of stigma and reputation concerns. J Cross-Cult Psychol. 2021;52(2):178–83.

[33] Smart R, Morral AR, Murphy JP, Jose R, Charbonneau A, Smucker S. The science of gun policy: A critical synthesis of research evidence on the effects of gun policies in the united States, fourth edition. Rand Health Q. 2024;12(1):3. 39664971 10.7249/rhq-12-01-03 PMC11630101

[34] Cai Z, Junus A, Chang Q, Yip PSF. The lethality of suicide methods: A systematic review and meta-analysis. J Affect Disord. 2022;300:121–9. 10.1016/j.jad.2021.12.054. 34953923 10.1016/j.jad.2021.12.054

[35] Barry R, Rehm J, de Oliveira C, Gozdyra P, Chen S, Kurdyak P. The relationship between rurality, travel time to care and death by suicide. BMC Psychiatry. 2023;23(1):345. 10.1186/s12888-023-04805-w. 37198612 10.1186/s12888-023-04805-w PMC10189916

[36] Poulson M, Jay J, Kenzik K, Torres C, Sanchez SE, Saillant N, Holena D, Galea S, Scantling D. Death by the minute: inequities in trauma care for victims of firearm violence. J Trauma Acute Care Surg. 2024;96(4):589–95. 10.1097/TA.0000000000004219. 37994476 10.1097/TA.0000000000004219 PMC10978261

[37] Wei R, Clay Mann N, Dai M, Hsia RY. Injury-based geographic access to trauma centers. Acad Emerg Med. 2019;26(2):192–204. 10.1111/acem.13518. 30019802 10.1111/acem.13518

[38] DuBois CM, Falls A, Serrano BN, Wagner HR, Tsai J, Elbogen EB. Socioeconomic correlates of suicidal ideation in military veterans: examining the interaction between homelessness and financial debt. Community Mental Health J 2024 Aug 7:1–0. 10.1007/s10597-024-01316-0 39110293

[39] Elbogen EB, Graziano RC, LaRue G, Cohen AJ, Hooshyar D, Wagner HR, Tsai J. Food insecurity and suicidal ideation: results from a National longitudinal study of military veterans. Archives Suicide Res. 2024;28(2):644–59. 10.1080/13811118.2023.2200795 PMC10636240 37165670

[40] Montgomery AE, Blosnich JR, deRussy A, Richman JS, Dichter ME, True G. Association between services to address adverse social determinants of health and suicide mortality among veterans with indicators of housing instability, unemployment, and justice involvement. Archives Suicide Res. 2024;28(3):860–76. 10.1080/13811118.2023.2244534 37565799

[41] United States Census Bureau, U.S. Department of Commerce. Poverty in States and Metropolitan Areas: 2023. [Internet]. 2024. [Available from: https://www2.census.gov/library/publications/2024/demo/acsbr-022.pdf

[42] U.S. Department of Agriculture, Economic Research Service. Food Security in the U.S. – Interactive Charts and Highlights. [Internet]. 2025. Available from: https://www.ers.usda.gov/topics/food-nutrition-assistance/food-security-in-the-us/interactive-charts-and-highlights#States

[43] Department of Housing and Urban Development, Office of Policy Development and Research. Annual Homelessness Assessment Report. [Internet]. 2024. Available from: https://www.huduser.gov/portal/datasets/ahar/2024-ahar-part-1-pit-estimates-of-homelessness-in-the-us.html

[44] U.S. Department of Agriculture, Economic Research Service. Food Security in the U.S. – Key Statistics & Graphics. [Internet]. 2025. Available from: https://www.ers.usda.gov/topics/food-nutrition-assistance/food-security-in-the-us/key-statistics-graphics

[45] U.S. Department of Agriculture, Economic Research Service. Employment & Education – Rural Employment and Unemployment. [Internet]. 2025. Available from: https://www.ers.usda.gov/topics/rural-economy-population/employment-education/rural-employment-and-unemployment

[46] Klimes-Dougan B, Mirza SA, Babkin E, Lanning C. Biased reporting of past self-injurious thoughts and behaviors: A literature review. J Affect Disord. 2022;308:596–606. 10.1016/j.jad.2022.04.073. 35429538 10.1016/j.jad.2022.04.073

[47] Hom MA, Stanley IH, Podlogar MC, Joiner TE Jr. Are you having thoughts of suicide?? Examining experiences with disclosing and denying suicide?al ideation. J Clin Psychol. 2017;73(10):1382–92. 10.1002/jclp.22440. 28085200 10.1002/jclp.22440

[48] Nguyen X-MT, Whitbourne SB, Li Y, Quaden RM, Song RJ, Nguyen H-NA, Harrington K, Djousse L, Brewer JVV, Deen J, Muralidhar S, Ramoni RB, Cho K, Casas JP, Tsao PS, Gaziano JM, the VA Million Veteran Program. Data Resource Profile: Self-reported data in the Million Veteran Program: survey development and insights from the first 850 736 participants. Int J Epidemiol. 2023;52(1):e1–e17. 10.1093/ije/dyac133. 35748351 10.1093/ije/dyac133

[49] Vogt DS, Tyrell FA, Bramande EA, et al. U.S. Military veterans' health and well-being in the first year after service. Am J Prev Med. 2020;58(3):352-360. 10.1016/j.amepre.2019.10.016 31902684 10.1016/j.amepre.2019.10.016

[50] Ammerman BA, Reger MA. Evaluation of prevention efforts and risk factors among veteran suicide decedents who died by firearm. Suicide Life-Threatening Behav. 2020;50(3):679–87. 10.1111/sltb.12618 32017233

[51] Mackowiak C, Eagan AE, Gerstel-Stanucci CM, Barnes KM, Brubaker CE, Fredritz KL, Rasnake LE, Landes SJ, Kearney LK, Miller MA. Expanding veteran suicide prevention: the role of community engagement and partnership coordinators. Psychol Serv. 2025. 10.1037/ser0000944. 40111858 10.1037/ser0000944

[52] SAMHSA, Governor’s. and Mayor’s Challenges to Prevent Suicide Among Service Members, Veterans, and their Families. [Internet]. 2024 [cited 2025 May 19]. Available from: https://www.samhsa.gov/smvf-ta-center/mayors-governors-challenges

