# Supplementary material for: Geospatial estimates of suicidal ideation and suicide attempt prevalence in the U.S. veteran population (2022)
Source: Inj Epidemiol. 2025 Jun 10;12:32. doi: 10.1186/s40621-025-00584-y (PMC12153138; doi:10.1186/s40621-025-00584-y)
Supplement: Supplementary file 1 — Supplementary Material 1 [file 40621_2025_584_MOESM1_ESM.docx]

Supplemental Table 2. Proportion of Veterans by State and Territory

| **Main Sample** |  |  |  |  |  |  |  |
| --- | --- | --- | --- | --- | --- | --- | --- |
| **State/Territory** |  |  |  |  |  |  |  |
|  | **Unweighted N** | **Unweighted %** | **Weighted N** | **Weighted %** | **95% CI** | **2022 State Population** | **Estimated % of Veterans in State Population** |
| Alabama | 243 | 1.40 | 307,771 | 1.97 | 1.82, 2.12 | 5,076,181 | 6.06 |
| Alaska | 207 | 1.19 | 58,903 | 0.38 | 0.33, 0.43 | 734,442 | 8.02 |
| Arizona | 368 | 2.12 | 404,631 | 2.59 | 2.43, 2.75 | 7,377,566 | 5.48 |
| Arkansas | 191 | 1.10 | 178,126 | 1.14 | 1.04, 1.24 | 3,047,704 | 5.84 |
| California | 1,093 | 6.28 | 1,238,900 | 7.94 | 7.54, 8.33 | 39,142,414 | 3.17 |
| Colorado | 282 | 1.62 | 334,143 | 2.14 | 1.92, 2.37 | 5,850,935 | 5.71 |
| Connecticut | 242 | 1.39 | 128,475 | 0.82 | 0.75, 0.90 | 3,617,925 | 3.55 |
| Delaware | 262 | 1.51 | 53,404 | 0.34 | 0.31, 0.37 | 1,020,625 | 5.23 |
| District of Columbia | 182 | 1.05 | 19,521 | 0.13 | 0.10, 0.15 | 676,725 | 2.88 |
| Florida | 1,098 | 6.31 | 1,191,122 | 7.63 | 7.38, 7.88 | 22,379,312 | 5.32 |
| Georgia | 495 | 2.85 | 593,017 | 3.8 | 3.61, 3.98 | 10,931,805 | 5.42 |
| Hawaii | 237 | 1.36 | 91,686 | 0.59 | 0.50, 0.68 | 1,440,359 | 6.37 |
| Idaho | 259 | 1.49 | 107,775 | 0.69 | 0.62, 0.76 | 1,944,299 | 5.54 |
| Illinois | 403 | 2.32 | 471,154 | 3.02 | 2.81, 3.23 | 12,621,821 | 3.73 |
| Indiana | 276 | 1.59 | 327,788 | 2.1 | 1.93, 2.27 | 6,844,545 | 4.79 |
| Iowa | 266 | 1.53 | 158,007 | 1.01 | 0.91, 1.11 | 3,202,820 | 4.93 |
| Kansas | 227 | 1.3 | 154,615 | 0.99 | 0.87, 1.11 | 2,937,324 | 5.26 |
| Kentucky | 205 | 1.18 | 237,091 | 1.52 | 1.35, 1.68 | 4,519,233 | 5.25 |
| Louisiana | 177 | 1.02 | 230,366 | 1.48 | 1.35, 1.61 | 4,593,687 | 5.01 |
| Maine | 260 | 1.49 | 94,584 | 0.61 | 0.55, 0.66 | 1,390,922 | 6.80 |
| Maryland | 314 | 1.81 | 299,644 | 1.92 | 1.79, 2.05 | 6,192,440 | 4.84 |
| Massachusetts | 203 | 1.17 | 230,941 | 1.48 | 1.33, 1.63 | 7,022,877 | 3.29 |
| Michigan | 438 | 2.52 | 427,866 | 2.74 | 2.59, 2.89 | 10,050,877 | 4.26 |
| Minnesota | 271 | 1.56 | 255,289 | 1.64 | 1.50, 1.77 | 5,721,621 | 4.46 |
| Mississippi | 216 | 1.24 | 158,877 | 1.02 | 0.93, 1.10 | 2,941,939 | 5.40 |
| Missouri | 289 | 1.66 | 340,280 | 2.18 | 2.03, 2.33 | 6,179,414 | 5.51 |
| Montana | 279 | 1.6 | 74,583 | 0.48 | 0.43, 0.53 | 1,122,095 | 6.65 |
| Nebraska | 245 | 1.41 | 104,127 | 0.67 | 0.61, 0.73 | 1,972,246 | 5.28 |
| Nevada | 211 | 1.21 | 184,137 | 1.18 | 1.10, 1.26 | 3,176,116 | 5.80 |
| New Hampshire | 225 | 1.29 | 85,080 | 0.55 | 0.49, 0.60 | 1,396,678 | 6.09 |
| New Jersey | 213 | 1.22 | 239,557 | 1.53 | 1.39, 1.68 | 9,295,227 | 2.58 |
| New Mexico | 262 | 1.51 | 123,784 | 0.79 | 0.73, 0.85 | 2,113,868 | 5.86 |
| New York | 510 | 2.93 | 549,068 | 3.52 | 3.33, 3.70 | 19,848,276 | 2.77 |
| North Carolina | 511 | 2.94 | 614,280 | 3.93 | 3.76, 4.10 | 10,564,320 | 5.81 |
| North Dakota | 230 | 1.32 | 43,769 | 0.28 | 0.25, 0.31 | 781,057 | 5.60 |
| Ohio | 504 | 2.9 | 583,166 | 3.74 | 3.52, 3.95 | 11,777,874 | 4.95 |
| Oklahoma | 225 | 1.29 | 241,684 | 1.55 | 1.43, 1.66 | 4,026,229 | 6.00 |
| Oregon | 253 | 1.45 | 235,734 | 1.51 | 1.38, 1.64 | 4,247,372 | 5.55 |
| Pennsylvania | 537 | 3.09 | 592,487 | 3.8 | 3.60, 3.99 | 12,984,990 | 4.56 |
| Puerto Rico | 236 | 1.36 | 60,596 | 0.39 | 0.31, 0.47 | 3,220,137 | 1.88 |
| Rhode Island | 208 | 1.2 | 46,881 | 0.3 | 0.27, 0.33 | 1,099,498 | 4.26 |
| South Carolina | 288 | 1.66 | 339,504 | 2.17 | 1.98, 2.37 | 5,287,935 | 6.42 |
| South Dakota | 271 | 1.56 | 56,232 | 0.36 | 0.33, 0.39 | 909,723 | 6.18 |
| Tennessee | 290 | 1.67 | 383,000 | 2.45 | 2.26, 2.64 | 7,062,217 | 5.42 |
| Texas | 1,029 | 5.92 | 1,330,571 | 8.52 | 8.23, 8.81 | 30,113,488 | 4.42 |
| Utah | 263 | 1.51 | 109,292 | 0.7 | 0.63, 0.77 | 3,391,011 | 3.22 |
| Vermont | 229 | 1.32 | 34,324 | 0.22 | 0.19, 0.25 | 648,142 | 5.30 |
| Virginia | 545 | 3.13 | 594,355 | 3.81 | 3.62, 4.00 | 8,683,414 | 6.84 |
| Washington | 371 | 2.13 | 466,058 | 2.99 | 2.75, 3.22 | 7,794,123 | 5.98 |
| West Virginia | 211 | 1.21 | 106,786 | 0.68 | 0.63, 0.74 | 1,774,122 | 6.02 |
| Wisconsin | 290 | 1.67 | 280,427 | 1.8 | 1.65, 1.94 | 5,903,975 | 4.75 |
| Wyoming | 256 | 1.47 | 37,900 | 0.24 | 0.22, 0.27 | 581,978 | 6.51 |
| **Pacific Islands Sample** | |  |  |  |  | **2020 Population Estimate*** |  |
| Guam | 485 | 87.7 | 8,264 | 86.84 | 82.73, 90.94 | 153,836 | 5.37 |
| CNMI | 41 | 7.41 | 817 | 8.58 | 5.25, 11.92 | 47,329 | 1.73 |
| American Samoa | 27 | 4.88 | 436 | 4.58 | 2.38, 6.78 | 49,710 | 0.88 |

*2022 population estimates are not available for Pacific Islands Territories. 2020 Census data are used instead.
