# Supplementary material for: Geospatial estimates of suicidal ideation and suicide attempt prevalence in the U.S. veteran population (2022)
Source: Inj Epidemiol. 2025 Jun 10;12:32. doi: 10.1186/s40621-025-00584-y (PMC12153138; doi:10.1186/s40621-025-00584-y)
Supplement: Supplementary file 2 — Supplementary Material 2 [file 40621_2025_584_MOESM2_ESM.docx]

Supplemental Table 1. Completed Survey Yield by State/Territory

| **State/Territory** | **Completed Surveys** | **Yield** |
| --- | --- | --- |
| Alabama | 242 | 16.6% |
| Alaska | 207 | 16.7% |
| Arizona | 364 | 18.9% |
| Arkansas | 195 | 15.3% |
| California | 1087 | 16.8% |
| Colorado | 283 | 18.5% |
| Connecticut | 249 | 19.5% |
| DC | 185 | 15.6% |
| Delaware | 266 | 21.0% |
| Florida | 1093 | 18.5% |
| Georgia | 497 | 18.1% |
| Hawaii | 241 | 19.5% |
| Idaho | 267 | 20.8% |
| Illinois | 407 | 17.2% |
| Indiana | 271 | 17.1% |
| Iowa | 262 | 20.4% |
| Kansas | 228 | 18.0% |
| Kentucky | 207 | 16.1% |
| Louisiana | 185 | 14.3% |
| Maine | 247 | 19.4% |
| Maryland | 312 | 20.5% |
| Massachusetts | 199 | 15.6% |
| Michigan | 432 | 20.2% |
| Minnesota | 269 | 20.9% |
| Mississippi | 206 | 16.0% |
| Missouri | 285 | 17.2% |
| Montana | 281 | 22.1% |
| Nebraska | 250 | 19.7% |
| Nevada | 207 | 16.1% |
| New Hampshire | 222 | 17.5% |
| New Jersey | 220 | 16.8% |
| New Mexico | 263 | 20.6% |
| New York | 503 | 17.1% |
| North Carolina | 512 | 18.4% |
| North Dakota | 231 | 18.4% |
| Ohio | 506 | 17.2% |
| Oklahoma | 225 | 17.1% |
| Oregon | 252 | 19.6% |
| Pennsylvania | 542 | 17.8% |
| Puerto Rico | 248 | 19.8% |
| Rhode Island | 210 | 16.9% |
| South Carolina | 289 | 18.7% |
| South Dakota | 270 | 21.4% |
| Tennessee | 286 | 16.0% |
| Texas | 1023 | 16.3% |
| Utah | 265 | 21.0% |
| Vermont | 235 | 18.8% |
| Virginia | 542 | 18.8% |
| Washington | 363 | 17.3% |
| West Virginia | 205 | 16.1% |
| Wisconsin | 363 | 17.3% |
| Wyoming | 253 | 20.0% |
| American Samoa | 36 | 16.4% |
| Guam | 493 | 19.5% |
| Northern Mariana Islands | 43 | 25.4% |
