# Supplementary material for: Geospatial estimates of suicidal ideation and suicide attempt prevalence in the U.S. veteran population (2022)
Source: Inj Epidemiol. 2025 Jun 10;12:32. doi: 10.1186/s40621-025-00584-y (PMC12153138; doi:10.1186/s40621-025-00584-y)
Supplement: Supplementary file 3 — Supplementary Material 3 [file 40621_2025_584_MOESM3_ESM.docx]

|  | Midwest (n=3,710) | | | | Northeast (n=2,627) | | | | South (n=6,482) | | | | West (n=4,341) | | | | Puerto Rico (n=236) | | | | Pacific Island Territories (n=553) | | | |
| --- | --- | --- | --- | --- | --- | --- | --- | --- | --- | --- | --- | --- | --- | --- | --- | --- | --- | --- | --- | --- | --- | --- | --- | --- |
|  | UW^1^ | Weighted | | | UW | Weighted | | | UW | Weighted | | | UW | Weighted | | | UW | Weighted | | | UW | Weighted | | |
|  | n | n | % | 95% CI | n | n | % | 95% CI | n | n | % | 95% CI | n | n | % | 95% CI | n | n | % | 95% CI | n | n | % | 95% CI |
| **Age** |  |  |  |  |  |  |  |  |  |  |  |  |  |  |  |  |  |  |  |  |  |  |  |  |
| 18-34 | 151 | 278,643 | 8.83 | 7.03, 10.63 | 97 | 174,565 | 8.87 | 6.68, 11.06 | 233 | 659,154 | 9.75 | 8.38, 11.12 | 176 | 374,248 | 10.91 | 8.70, 13.13 | 3 | 252 | 0.42 | 0.00,0.94 | 29 | 1,500 | 16.05 | 10.28, 21.82 |
| 35-49 | 434 | 576,217 | 18.26 | 16.22, 20.30 | 278 | 339,352 | 17.24 | 14.88, 19.61 | 767 | 1,289,367 | 19.07 | 17.68, 20.45 | 563 | 666,216 | 19.43 | 17.39, 21.47 | 23 | 13,179 | 22.22 | 11.12, 33.33 | 103 | 2,237 | 23.94 | 18.33, 29.56 |
| 50-64 | 1,046 | 959,625 | 30.41 | 28.40, 32.42 | 722 | 597,053 | 30.34 | 27.96, 32.72 | 1,849 | 2,081,187 | 30.77 | 29.40, 32.15 | 1,101 | 931,275 | 27.16 | 25.28, 29.03 | 64 | 21,243 | 35.82 | 24.66, 46.99 | 203 | 3,143 | 33.64 | 26.74, 40.54 |
| 65+ | 2,012 | 1,340,843 | 42.49 | 40.53, 44.46 | 1,474 | 856,944 | 43.55 | 41.13, 45.96 | 3,492 | 2,732,953 | 40.41 | 39.11, 41.72 | 2,428 | 1,457,516 | 42.50 | 40.58, 44.42 | 141 | 24,629 | 41.53 | 29.79, 53.27 | 204 | 2,464 | 26.37 | 13.92, 38.81 |
| **Gender** |  |  |  |  |  |  |  |  |  |  |  |  |  |  |  |  |  |  |  |  |  |  |  |  |
| Man | 2,967 | 2,857,589 | 90.38 | 89.80, 90.96 | 2,118 | 1,791,868 | 90.71 | 89.93, 91.49 | 5,133 | 5,919,036 | 87.30 | 86.79, 87.80 | 3,410 | 3,045,302 | 89.02 | 88.35, 89.69 | 187 | 56,408 | 93.28 | 90.52, 96.03 | 483 | 8,130 | 86.76 | 82.75, 90.78 |
| Woman | 662 | 290,805 | 9.20 | 8.69, 9.70 | 448 | 166,603 | 8.43 | 7.88, 8.99 | 1,198 | 825,000 | 12.17 | 11.71, 12.63 | 831 | 349,991 | 10.23 | 9.64, 10.82 | 41 | 3,201 | 5.29 | 3.19, 7.40 | 58 | 1,240 | 13.24 | 9.22, 17.25 |
| Transgender | 12 | 11,897 | 0.38 | 0.09, 0.67 | 9 | 8,862 | 0.45 | 0.08, 0.81 | 13 | 15,503 | 0.23 | 0.05, 0.40 | 14 | 15,034 | 0.31 | 0.11, 0.51 | 2 | 117 | 0.19 | 0.00, 0.54 | 0 |  |  |  |
| Non-binary or Other | 6 | 1,594 | 0.05 | 0.00, 0.10 | 8 | 8,024 | 0.41 | 0.00, 0.88 | 16 | 20,889 | 0.31 | 0.13, 0.49 | 13 | 10,613 | 0.31 | 0.11, 0.51 | 2 | 748 | 1.24 | 0.00, 3.05 | 0 |  |  |  |
| **Race (mutually exclusive)** |  |  |  |  |  |  |  |  |  |  |  |  |  |  |  |  |  |  |  |  |  |  |  |  |
| White | 2,965 | 2,811,045 | 89.93 | 89.09, 90.76 | 2,167 | 1,704,810 | 87.63 | 86.40, 88.85 | 4,667 | 5,086,322 | 76.42 | 75.42, 77.41 | 3,276 | 2,754,377 | 83.22 | 81.67, 84.77 | 158 | 39,463 | 81.68 | 71.98, 91.39 | 95 | 1,489 | 16.04 | 11.90, 20.18 |
| Black | 360 | 167,407 | 5.36 | 4.78, 5.93 | 214 | 134,701 | 6.92 | 5.94, 7.91 | 1,109 | 1,071,037 | 16.09 | 15.17, 17.02 | 223 | 147,809 | 4.47 | 3.68, 5.25 | 13 | 3,127 | 6.47 | 0.52, 12.43 | 19 | 314 | 3.38 | 1.73, 5.04 |
| AI/AN | 69 | 18,592 | 0.59 | 0.32, 0.86 | 11 | 5,786 | 0.30 | 0.00, 0.64 | 49 | 45,031 | 0.68 | 0.43, 0.92 | 82 | 36,784 | 1.11 | 0.72, 1.50 | 0 |  |  |  | 3 | 58 | 0.62 | 0.00, 1.38 |
| Asian | 32 | 13,644 | 0.44 | 0.18, 0.69 | 28 | 18,350 | 0.94 | 0.44, 1.44 | 40 | 50,705 | 0.76 | 0.43, 1.09 | 155 | 109,885 | 3.32 | 2.67, 3.97 | 1 | 498 | 1.03 | 0.00, 3.05 | 57 | 922 | 9.94 | 6.69, 13.19 |
| Native Hawaiian | 0 |  |  |  | 2 | 1,501 | 0.08 | 0.00, 0.23 | 2 | 2,796 | 0.04 | 0.00, 0.10 | 9 | 5,885 | 0.18 | 0.05, 0.31 | 0 |  |  |  | 1 | 15 | 0.17 | 0.00, 0.49 |
| Pacific Islander | 6 | 1,374 | 0.04 | 0.01, 0.08 | 2 | 96 | 0.01 | 0.00, 0.01 | 13 | 11,337 | 0.17 | 0.05, 0.29 | 23 | 25,185 | 0.76 | 0.15, 1.38 | 0 |  |  |  | 280 | 4,257 | 45.87 | 37.29, 54.46 |
| Other | 13 | 6,860 | 0.22 | 0.06, 0.37 | 6 | 6,465 | 0.33 | 0.00, 0.69 | 30 | 31,369 | 0.47 | 0.24, 0.70 | 25 | 41,005 | 1.24 | 0.13, 2.35 | 5 | 789 | 1.63 | 0.00, 3.76 | 0 |  |  |  |
| Multi-racial | 144 | 106,993 | 3.42 | 2.67, 4.18 | 115 | 73,814 | 3.79 | 2.83, 4.76 | 324 | 357,488 | 5.37 | 4.65, 6.09 | 312 | 188,838 | 5.71 | 4.88, 6.53 | 18 | 4,437 | 9.18 | 1.51, 16.85 | 80 | 2,225 | 23.98 | 10.83, 37.12 |
| **Rurality** |  |  |  |  |  |  |  |  |  |  |  |  |  |  |  |  |  |  |  |  |  |  |  |  |
| Rural | 1,253 | 998,179 | 31.26 | 29.24, 33.28 | 660 | 369,155 | 18.50 | 16.69, 20.31 | 1,372 | 1,386,407 | 20.19 | 19.03, 21.35 | 990 | 523,240 | 15.11 | 13.83, 16.40 | 0 |  |  |  | * |  |  |  |
| Urban | 2,446 | 2,195,084 | 68.74 | 66.72, 70.76 | 1,956 | 1,626,310 | 81.50 | 79.69, 83.31 | 5,098 | 5,480,457 | 79.81 | 78.65, 80.97 | 3,339 | 2,939,217 | 84.89 | 83.60, 86.17 | 235 | 60,532 | 100.00 |  |  |  |  |  |
| **Time since Separation** |  |  |  |  |  |  |  |  |  |  |  |  |  |  |  |  |  |  |  |  |  |  |  |  |
| Less than 4 years | 96 | 119,249 | 3.72 | 2.63, 4.82 | 65 | 72,126 | 3.60 | 2.66, 4.55 | 183 | 326,820 | 4.75 | 3.97, 5.53 | 101 | 171,156 | 4.94 | 3.33, 6.55 | 8 | 2,572 | 4.24 | 0.05, 8.44 | 47 | 1,214 | 12.75 | 8.50, 17.01 |
| 4-9 years | 196 | 268,535 | 8.38 | 6.78, 9.99 | 133 | 147,516 | 7.37 | 5.59, 9.15 | 394 | 697,527 | 10.14 | 8.90, 11.38 | 258 | 361,694 | 10.43 | 8.39, 12.48 | 9 | 5,652 | 9.33 | 2.50, 16.15 | 57 | 1,448 | 15.21 | 10.36, 20.07 |
| 10+ years | 3,418 | 2,814,937 | 87.89 | 86.10, 89.69 | 2,429 | 1,781,755 | 89.03 | 87.14, 90.91 | 5,905 | 5,854,736 | 85.11 | 83.84, 86.36 | 3,982 | 2,934,675 | 84.63 | 82.43, 86.83 | 219 | 52,371 | 86.43 | 78.66, 94.19 | 449 | 6,855 | 72.03 | 65.39, 78.68 |

**Supplemental Table 3. Unweighted and Weighted Participant Characteristics by Region (n=17,949)**

^1^UW= Unweighted; *Rurality was categorized based on RUCA codes. The Pacific Islands Territories do not have assigned RUCA codes, and thus rurality designation is not provided for the PI Territories sample.
